# SATB2 dysregulation generates a novel circular RNA and drives *KRAS*-like transcriptional reprogramming and transformation-associated phenotypes

**DOI:** 10.1186/s12964-026-03021-9

**Published:** 2026-06-24

**Authors:** Rebekah Eleazer, Smitha George, Luke J. Shoemaker, Wesley N. Saintilnord, Richard N. Cassidy, Andrew Pyman, Kin Lau, Molly T. Soper-Hopper, Hyoungjoo Lee, Darrell P. Chandler, Yvonne N. Fondufe-Mittendorf

**Affiliations:** 1 Department of Molecular and Cellular Biochemistry, University of Kentucky, Lexington, KY 40536 USA; 2 Department of Epigenetics, Van Andel Institute, Grand Rapids, MI 49503 USA; 3 Bioinformatics & Biostatistics Core, Van Andel Institute, Grand Rapids, MI 49503 USA; 4 Mass Spectrometry Core, Van Andel Institute, Grand Rapids, MI 49503 USA

## Abstract

**Background:**

Even though epigenetic factors contribute to oncogenesis, most human cancer models still assume that disease originates from driver DNA mutations. Thus, it is still unclear if non-genetic mechanisms are sufficient to trigger malignant transformation. Special AT-rich binding protein 2 (SATB2) is a chromatin organizer that brings distal DNA elements into close proximity, thus remodeling chromatin structures to reprogram cell-specific and/or developmentally sensitive gene networks.

**Methods:**

Here, we chronically exposed human bronchial lung epithelial cells to ≤ 2 µM inorganic arsenic, and then used biochemical, molecular, and phenotypic assays to understand how changes in *SATB2* expression and chromatin structure relate to oncogenic transformation.

**Results:**

We discovered that *SATB2* generates a co-expressed *circ3915* RNA that can be translated into a peptide that co-localizes with SATB2 in and around the nuclear membrane. Ectopic *SATB2* or *circ3915* expression rearranged global chromatin accessibility, generated *KRAS*- and *NFE2L2*-like oncogenic gene expression patterns, and induced oncogenic phenotypes and KRAS-like transcriptional programs in lung epithelial cells without iAs exposure or engineered driver mutations. *SATB2* and *circ3915* transcripts persisted through the epithelial-to-mesenchymal transition and were co-regulated in human LUAD and LUSC tumors and adjacent normal tissue.

**Conclusions:**

This study shows that transcriptional programs associated with oncogenic pathways can be activated in differentiated mammalian cells without predisposing mutations in oncogenes or epigenetic regulators.

**Graphical Abstract:**

**Figure:**
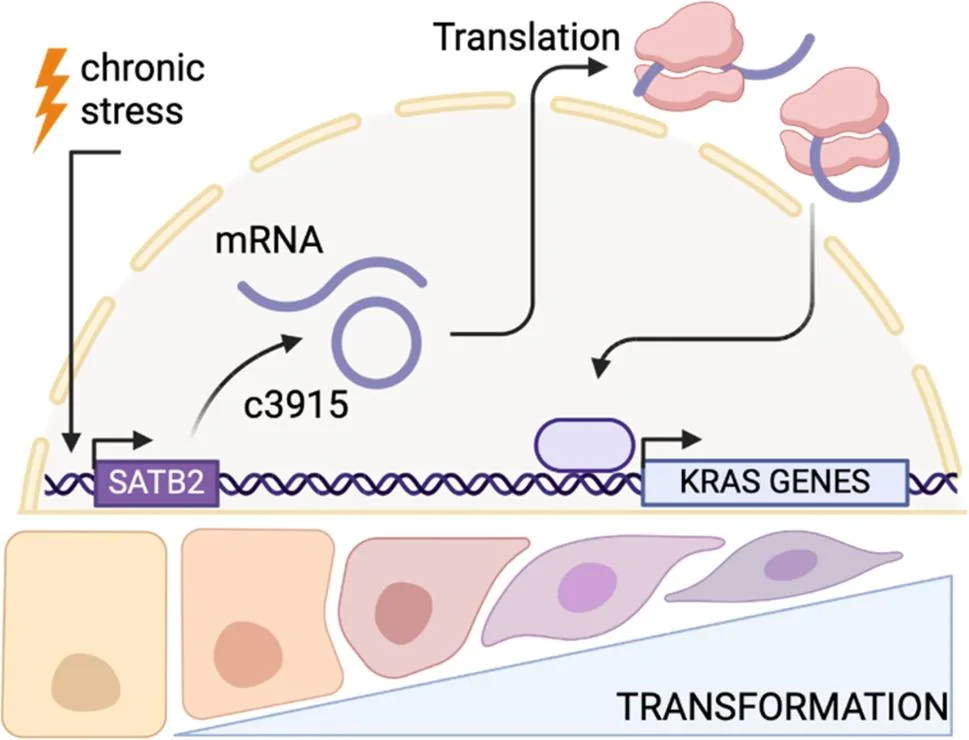

**Supplementary Information:**

The online version contains supplementary material available at https://doi.org/10.1186/s12964-026-03021-9.

## Introduction

Cancer is usually viewed as a genetic disease arising from DNA mutations [1, 2], but we now appreciate that cancer is also caused by DNA and histone modifications that alter gene expression patterns [3–5]. Indeed, cancer genomes exhibit substantial changes across multiple layers of epigenetic regulation [6] and can be heavily influenced by environmental factors [7].

Epigenetics implies that cancer can arise independent of DNA mutations, yet most studies into epigenetic dysregulation and oncogenesis still focus on DNA mutations in those genes/factors that regulate histone post-translational modifications, DNA methylation, microRNA expression, or genome folding [8]. And while a recent study showed that transient, Polycomb-mediated silencing can trigger an irreversible switch to a cancer cell fate in *Drosophila* [9], it is still unclear if non-genetic mechanisms contribute to oncogenic reprogramming in mammalian systems.

Three-dimensional (3D) chromatin structure is an often-overlooked epigenetic mechanism that can regulate gene expression [10, 11] and contribute to oncogenesis [12–14]. 3D chromatin structures are often influenced by environmental factors, as is the case for the CCCTC-binding factor (CTCF) [15] and its thousands of CTCF-binding sites [16–19]. For example, inorganic arsenic (iAs) selectively inhibits CTCF binding and generates new 3D chromatin structures that drive (onco)gene expression patterns [20]. iAs exposure also causes other oncogenic epigenetic changes [21–25] that can occur genome-wide or gene-specific manner [20, 22, 26], thus altering chromatin accessibility at cis-regulatory elements [27–30]. In some respects, then, iAs exposure and related toxicology models provide novel mechanistic insights into epigenetic regulation, chromatin structure, and oncogenesis that might otherwise be missed in mutation-associated cancer models.

Special AT-rich binding protein 2 (SATB2) is a 3D chromatin organizer that operates independently of and in cooperation with CTCF [31]. It regulates gene expression [32–34] by binding to the nuclear matrix and regulatory DNA elements, thereby bringing distal DNA elements into close spatial proximity [32, 35]. These SATB2-associated chromatin architectures serve as platforms for chromatin remodeling complexes [36–38], enabling the coordinated regulation of cell-specific and/or developmentally responsive gene networks in response to external cues [39–43]. In most adult tissues, *SATB2* expression is tightly controlled and is typically suppressed by microRNAs (miRNAs), small (~ 20 nt) endogenous RNAs that bind target transcripts to promote degradation or inhibit translation [44]. Disrupting this SATB2 mRNA–miRNA regulatory axis is associated with multiple cancers [45], including iAs-induced transformation [46, 47]. In some cancers, *SATB2*-targeted miRNAs are themselves negatively regulated by a circular RNA that is generated from the *SATB2* gene (e.g., circSATB2; circBase ID 0008928) [48–51]. Like other circular RNAs [52–59], circSATB2 arises from head-to-tail splicing of *SATB2* exons [48] and acts as a “sponge” for SATB2-targeted miRNAs or RNA-binding proteins to post-transcriptionally regulate *SATB2* translation [48–51]. Thus, SATB2 expression, translation, and function involve multiple layers of regulation, and disrupting any one of these layers can alter gene expression patterns to promote oncogenesis.

Here, we identify a novel SATB2-derived circular RNA (*circ3915*) originating from exons 3–6 of the SATB2 gene that is co-expressed with SATB2 mRNA upon iAs exposure. SATB2 and *circ3915* dysregulation are associated with changes in chromatin accessibility, induction of KRAS- and NFE2L2-like gene expression patterns, and the emergence of oncogenic phenotypes and transcriptional states in the absence of *KRAS* mutations. These findings support a model in which epigenetic dysregulation can engage oncogenic transcriptional programs without introducing defined driver mutations.

## Methods

### Patient samples

All de-identified patient samples used in this study were obtained from the Van Andel Institute Pathology and Biorepository Core (RRID: SCR_022912) and were histologically confirmed as either lung adenocarcinoma (LUAD) or lung squamous cell carcinoma (LUSC).

Lung tissues were snap-frozen in liquid nitrogen, pulverized, and RNA purified using a Qiagen RNeasy Plus Universal Mini Kit.

### Cell growth conditions and iAs-induced cell transformation

BEAS-2B cells were cultured in Dulbecco’s Modified Eagle’s Medium (DMEM) supplemented with 10% fetal bovine serum (v/v) and 100 U/mL penicillin-streptomycin. Cells were grown to ~ 80% confluency in a humidified chamber at 37 °C, 5% CO_2_. The Human Bronchial Epithelial cell line (16HBE) was a gift from Dr. Shaun McCullough [60] and grown on tissue culture flasks coated with type I collagen. Cells were cultured in Modified Eagle’s Medium (Advanced MEM (Gibco)) supplemented with 2% fetal bovine serum (v/v), 1% GlutaMAX, and 0.2% penicillin/streptomycin. All cells were routinely tested for mycoplasma every month and were confirmed negative.

Thereafter, cells were treated with iAs (as NaAsO_2_) in two ways. For “rapid” cell transformation, we sub-cultured and attached cells overnight and then treated cells with 2.0 µM until they reached ~ 80% confluence. This process was repeated every 3 or 4 days for 12 weeks. We also transformed cells with a “two-hit” and time-dependent in vitro iAs exposure system described by Rea et al. [61]. In this case, cells were treated with freshly diluted 0.5 µM iAs at each cell passage for 17 weeks. At the end of 17 weeks, cells were then exposed to 2.0 µM iAs for another 10 weeks. The time-matched controls for the “second hit” were cells that continued to be exposed to 0.5 µM iAs. Every experiment with both exposure models included time-matched vehicular controls, and we monitored all cells for altered morphology throughout the exposure period. The specific exposure model used for each experiment is indicated in the corresponding figure legend.

### *SATB2* and *circ3915* siRNA knockdown

siRNAs used to knock down *SATB2* and *circ3915* RNA are itemized in Table S1. To knock down *SATB2* mRNA, we used pre-validated ON-TARGETplus siRNAs from Horizon. Three of these siRNAs did not overlap with any circ3915 sequence (probes SATB2-1, -2, and − 3) and were therefore pooled for linear *SATB2* mRNA knockdown. The remaining ON-TARGET siRNA (SATB2-Circular+Linear) was used to simultaneously knock down *SATB2* and circ3915 RNAs (Fig S1A). We also used the circular RNA interactome resource (https://circinteractome.nia.nih.gov/index.html) to design three siRNAs specifically targeting the circ3915 back-splice junction (circ3915-1, -2, and − 3). The ON-TARGET non-targeting siRNA was used as a control for all experiments.

### *SATB2* and *circ3915* shRNA knockdown

Pre-designed and validated shRNAs from Millipore Sigma were used for stable *SATB2* knock-down (Table S2; Fig S1B). The reported knockdown efficiency for the *SATB2* circular and linear probe (shSATB2-2) is 84% (TRCN0000020687-predesigned shRNA), and our experimental knockdown efficiency ranged from 72 to 82% (see knockdown results). The *circ3915* shRNA was based on the *circ3915* siRNAs, above. Arsenic-transformed cells were stably transduced with each shRNA (lentivirus U5 promoter-shRNA; CMV-GFP vector) by the University of Michigan Vector Core Laboratory.

### *SATB2* and *circ3915* expression vectors

Full-length *SATB2* mRNA was amplified by PCR from plasmids that were kindly provided by the Costa Lab [47]. PCR products were purified with a Monarch PCR and DNA Cleanup Kit and assembled in the pcDNA3.1(+) vector using the NEBuilder^®^ HiFi DNA Assembly Cloning Kit to generate SATB2 GFP-tagged constructs with the GFP tag at the SATB2 C-terminus. For circ3915, we cloned the spliced exons of circ3915 (*SATB2* exons 3–6) into the pcDNA3.1(+) CircRNA Mini Vector. This vector surrounds the insert with splice sites and flanking *Alu*-containing intronic sequences for efficient back-splicing [62] (Fig S1C). Gibson assembly was then used to generate circ3915 constructs with the 3×FLAG-tag at the C-terminus of the putative circ3915 peptide. To generate a translation-deficient circ3915 construct, the canonical start codon ATG was mutated to GTG. All tags on circular constructs were inserted downstream of the back-splice junction but upstream of putative start codons to ensure that tags were only translated from back-spliced transcripts. The sequence of all expression constructs was confirmed by Sanger sequencing.

### Transient transfection of human cell lines

For transient *SATB2* and *circ3915* expression, plasmids (or empty vectors) were transfected into non-treated BEAS-2B cells; for siRNA knockdown experiments, siRNAs were transfected into iAs-transformed BEAS-2B cells. In both cases, we transfected cells with Lipofectamine 3000 reagent in Opti-MEM™ I Reduced Serum Medium according to the manufacturer’s instructions. Cells were incubated for 24–48 h after transfection and then collected for downstream analyses. RNA expression was confirmed by RT-qPCR and NanoString, and protein expression was confirmed by immunoblots.

### Lentiviral production, transduction, and selection

For stable *SATB2* and *circ3915* expression from lentiviruses, we used SATB2 with GFP tag and circ3915-3×FLAG tag/HiBiT tag (Fig S1D). These plasmids were then subcloned into lentiviruses by the University of Michigan Vector Core. Briefly, lentivirus packaging vectors psPAX2 and pC1-VSVG were co-transfected with proviral plasmid at approximately a 1:1:2 ratio, respectively, using standard polyethylenimine (PEI) precipitation methods (MW 2500). Three µg PEI per 1 µg DNA were incubated in Opti-MEM at room temperature for 20 min, and the mixture was then added to fresh DMEM supplemented with 10% FBS (v/v). The DNA/PEI-containing media was then added to HEK-293T cells to propagate the lentivirus. Supernatants were collected after 72 h and spun at 800 x g for 10 min to remove cellular debris. The viral supernatant was then concentrated on a 0.45 μm HV-Durapore Stericup 13,000 rpm on a Beckman Avanti J-E centrifuge at 4 °C for 4 h and resuspended at 10X the original concentration in DMEM (~ 1 × 10^7^ TU mL^− 1^). The concentrated lentivirus was stored in aliquots at -80 °C until use.

Lentivirus transduction and FACS sorting were performed at the University of Michigan Vector Core and Flow Cytometry Core, respectively. Briefly, BEAS-2B cells were transduced with several lentivirus titers (between 0.5-1x; MOI ≤ 1) in multiple wells of a 6-well plate. After 48 h, cells were either treated with 1 µg mL^− 1^ puromycin for 5 days (or until non-transduced cells were dead) or subjected to GFP or RFP sorting on a Sony SH800 Cell Sorter.

### Split luciferase translation assay

BEAS-2B cells containing circ3915-HiBiT or empty vector were detached by incubating in 0.5% trypsin for 5 min, washed in PBS, and then re-suspended in PBS. Cells were diluted 1:10 in PBS, and an equal volume of trypan blue was added to the diluted cells. Cells were then loaded onto a slide and counted on a Countess II FL automated cell counter (Invitrogen). The concentration of the stock cell suspension was then calculated, and an appropriate amount of PBS was added to the stock cell suspension to achieve ~ 10,000 cells per 100 µL. 100 µL of cell suspension (~ 10,000 cells) was then added to replicate wells (*n* = 6 per cell group) in a white opaque 96-well plate. An additional six wells were loaded with 100 µL of room temperature PBS to measure background luminescence. Next, an equal volume of Nano-Glo Luciferase Assay Reagent was added to each well. To ensure complete lysis and sufficient mixing, the plate was shaken on an orbital shaker for 30 s and then incubated for 10 min at room temperature. Relative luminosity (RLU) was measured on a Biotek Synergy HT Multi-Mode Microplate Reader with an integration time of 1.25 s. The RLU from PBS-only wells was subtracted from the RLU values from cell-containing wells in the same row, and relative luminescence was calculated as the fold-change of circ3915-HiBiT expressing cells relative to empty vector cells. Each experiment was performed in biological triplicate.

### Luciferase cell proliferation assay

We used the CellTiter-Glo ATP Assay to measure cell proliferation 72 h after seeding. Non-treated cells were the negative control for iAs-transformed and KRAS-G12V/TP53 BEAS-2B cells; iAs-transformed cells treated with non-targeting siRNAs were the negative control for iAs-transformed cells treated with siRNAs; and cells transfected with empty vector were the negative controls for cells stably expressing *SATB2*, *circ3915*, and circular + linear *SATB2* (see Results). Luminescence was quantified on a BioTek Synergy plate reader. Background luminescence was subtracted from the luminescence of the samples and background-corrected using the luminescence readings from cells that did not receive the knockdown treatment (above). Each experiment was performed three times with six technical replicates each.

### Trans-well migration assay

We used an in vitro trans-well assay to measure cell migratory potential. BEAS-2B cells were grown in complete media (DMEM + 10% FBS + penicillin-streptomycin), and then starved in blocking media (DMEM + 0.1% BSA) for 24 h. Starved cells were collected with trypsin, and ~ 2 × 10^4^ cells (in 100 µL) were seeded on the upper compartment of each trans-well insert (Greiner Bio-One ThinCert, 8 μm pore) that had been treated for 30–60 min with a coating solution (1x PBS, 15 µg mL^− 1^ collagen, 0.1 N NaOH). The lower chamber contained 600 µL of a migration-inducing media (DMEM, 0.1% BSA, 20 ng µL^− 1^ EGF). Cells were incubated for 24 h, and those cells that had migrated through the insert were washed in 1x PBS, fixed in methanol for 15 min, stained for 15 min in (1x PBS, 1% TritonX-100, 10 µg mL^− 1^ DAPI), and washed in 1x PBS. Stained cells were then imaged on an Olympus microscope at 40x magnification and counted using the cellSens counting module. To compare the effect of a specific treatment on cell migration, we normalized all cell counts to non-treated control cells.

### Total RNA extraction

Unless otherwise specified, total RNA was extracted from washed and pelleted cells using the Zymo Quick-RNA Miniprep Kit per the manufacturer’s protocol, and eluted in RNase-free water. RNA concentrations were measured by spectrophotometry, and RNA integrity was assessed on an Agilent Bioanalyzer before downstream RT-qPCR, NanoString, and RNA-seq analysis. Purified RNA was stored at -80 °C.

### Polysome fractionation and RNA extraction

Samples were prepared using previously published methods [63, 64], with some modifications to accommodate experimental needs. iAs-transformed BEAS-2B cells or BEAS-2B cells expressing the circ3915-HiBiT RNA were seeded in 15 cm^2^ plates (three replicate plates per condition) and grown to 70–80% confluency in DMEM + 10% FBS + penicillin-streptomycin (complete media). Cells were then treated with 1 mM puromycin for 3 h, after which the media was replaced with complete media + 100 µg mL^− 1^ cycloheximide for 5 min at 37 °C. Cells were then washed twice with 10 mL of ice-cold PBS + 100 µg mL^− 1^ cycloheximide and collected by centrifugation at 250 x g for 5 min at 4 °C. Cell pellets were resuspended in 450 µL hypotonic buffer (140 mM NaCl, 1.5 mM MgCl_2_, 10 mM Tris pH 7.5, 0.5% NP40, 100 µg mL^− 1^ cycloheximide, 1x HALT Protease Inhibitor Cocktail, 2.5 mM DTT, and 1 U µL^− 1^ RNAseOut), and vortexed for 5 s. We then added 25 µL of 10% Triton X-100 and 25 µL of 10% sodium deoxycholate to the resuspended cells. Nuclei were recovered by centrifugation at 16,000 x g for 7 min at 4 °C. The supernatant was collected and kept on ice, and 10% of the total volume (50 µL) was immediately added to a 1.5 mL microfuge tube containing 750 µL TRIzol reagent and flash-frozen in liquid nitrogen. The OD_260nm_ was measured and cytoplasmic lysates normalized to an equivalent OD. Lysates were loaded dropwise onto 10 mL 5%-50% sucrose gradients, and the loaded sucrose gradients were centrifuged at 36,000 rpm for 2 hrs at 4 °C in a SW41Ti rotor. Fractions were collected (at room temperature) from each gradient using a BioComp automated piston gradient fractionator. The resulting fractions were collected in 750 µL TRIzol, flash frozen in liquid nitrogen, and stored at -80 °C until RNA extraction.

RNA was extracted from each fraction by first mixing 0.2 mL chloroform per 1 mL of TRIzol and separating the phases by centrifugation at 12,000 x g for 15 min at 4 °C. The aqueous phase (~ 0.5 mL) was transferred to a new tube, mixed with 0.5 mL isopropanol, and incubated for 10 min at 4 °C. Precipitated RNA was collected by centrifugation at 12,000 x g at 4 °C, washed twice with 75% ethanol, air-dried, and resuspended 10–20 µL nuclease-free water. Purified RNA was then treated with DNAse I according to the manufacturer’s instructions. The distribution and abundance of *SATB2* and *circ3915* RNA molecules across the polysome gradient were then analyzed by RT-qPCR (below). 

### *SATB2* and *circ3915* RT-qPCR

RT-qPCR primers are listed in Table S3. The *circ3915* RNA consists of four *SATB2* exons and is generated from *SATB2* mRNA by back-splicing exons 3 and 6 (Fig S1E). We therefore used this backsplice junction to specifically detect *circ3915*. RT-qPCR was performed on a BioRad CFX96 Real-Time system using a thermal cycling program consisting of [95 °C for 5 min; and 40 cycles of 30 s at 95 °C, 30 s at 60 °C, and 10 s at 72 °C]. We also instituted a melting curve step based on the instrument’s temperature gradient settings. C_t_ values were normalized against the total RNA input or the geometric mean of three housekeeping genes (*GAPDH*, *RPII*, and *TBP*), and normalized values were used to calculate fold-change relative to control samples.

### Microarray hybridization and analysis

Total RNA from each sample was quantified using a NanoDrop ND-1000 (Thermo Fisher Scientific), and then sent to Arraystar (Rockville, MD) for circRNA analysis. Briefly, total RNA was first digested with RNase R to enrich for circRNAs.

The enriched circRNAs were amplified and labeled using the Arraystar Super RNA labeling kit, purified with a Qiagen RNeasy Mini Kit, and quantified on a NanoDrop ND-100. Then, 1 µg of the amplified circRNAs were fragmented by adding 5 µL of 10X blocking agent and 1 µL of 25x fragmentation buffer, and incubating at 60 °C for 30 min. Fragmented RNAs were then diluted with 25 µL of 2x hybridization buffer, and the entire solution (50 µL) was added to an Arraystar Human Circular RNA Array (#AS-S-CR-H-V2.0). Microarrays were incubated for ~ 17 h at 65 °C in an Agilent hybridization oven, washed, fixed, and then scanned on an Agilent G2505C scanner. The resulting images were processed with the Agilent feature extraction software (v11.0.1.1; Agilent Technology, Inc.). Expression analysis was conducted by applying quantile normalization and processing the normalized data with the R *limma* package (https://www.r-project.org/). A low-intensity filtering step was implemented, retaining only circRNAs surpassing a specific threshold. Differentially expressed circRNAs were defined as those exhibiting ≥ 1.2 fold-change and a significance level of *p* < 0.05. Volcano plots were generated in R-3.3.1 *gplots*, and we used the *heatmap2* function to generate hierarchical clusters and visualize variable circRNA-expression patterns among samples. Data conversion was conducted based on Z-score normalization.

### RNA-seq

Total RNA-seq libraries were prepared and sequenced by the Van Andel Genomics Core. Briefly, ribosomal RNAs were depleted from 500 ng of total RNA using a QIAseq FastSelect –rRNA HMR Kit. Libraries were then prepared with a KAPA RNA HyperPrep Kit, making sure to shear RNAs to the recommended 300–400 nt length before converting RNA to cDNA. cDNA fragments were ligated to IDT for Illumina TruSeq UD Indexed adapters, and indexed libraries were amplified via PCR. Library quality and quantity were then evaluated using Agilent DNA High Sensitivity chips, the Promega QuantiFluor dsDNA System, and Kapa Illumina Library Quantification qPCR assays. The individually indexed libraries were then pooled, and 50 bp paired-end sequencing was performed on an Illumina NovaSeq6000 sequencer to an average depth of 150 M raw paired reads per transcriptome. Base calling was done with Illumina RTA3, then demultiplexed and converted to FastQ format with Illumina Bcl2fastq v2.20.0.

Adapter sequences and low-quality bases were removed with TrimGalore v0.6.10 (https://github.com/FelixKrueger/TrimGalore) using the parameters ‘--paired -q 20’. Trimmed reads were aligned to the hg38 reference genome (GENCODE release 33) [65], and counted using STAR v2.7.10a [66] with the parameters ‘--twopassMode Basic --quantMode GeneCounts’. Raw counts were then converted to variance stabilizing transformation (VST) counts. Principal component analysis (PCA) was performed with the top 10,000 genes with the highest variance, using the *vst()* function in DESeq2 v1.38.3 with ‘blind=FALSE’ [67]. We used DESeq2 and the design ‘~ group’ to identify differentially expressed genes (DEGs). Pairwise contrasts were tested for significance at *p*_*adj*_ ≤ 0.01. Log-fold changes were shrunk using the *lfcShrink* function with the parameters ‘type="ashr"’ [68]. Gene set enrichment analysis (GSEA) was conducted using clusterProfiler v4.6.2 [69] with the parameter ‘eps = 0.0’. Genes were ranked based on the shrunken log fold changes and a significance cutoff of *p*_*adj*_ ≤ 0.05. MSigDB annotations were retrieved using msigdbr v7.5.1, and summary plots were created with enrichPlot v1.18.4.

### NanoString analyses

We used direct digital counting with custom NanoString codesets (Fig S1F, Table S4) to verify differentially expressed circular (microarray) and linear (RNA-seq) transcripts. For circRNA expression, we designed capture and reporter probes for the backsplice junction of the top candidate circRNAs. In cases where circRNAs shared an exon at the backsplice junction, protector probes were designed to prevent cross-hybridization. For linear RNAs, capture and reporter probes were designed to target regions overlapping linear (consecutive) splice junctions. To detect linear transcripts arising from those genes that also produce circRNAs, probes were designed against regions that did not correspond with candidate circRNA sequences. We also included probes for 12 reference genes that are known to have high, medium, or low intrinsic expression levels. Total RNA isolated from experimental and control cells was subjected to nCounter™ SPRINT (NanoString Technologies) analysis according to the manufacturer’s instructions. Background subtraction, normalization, quality control, and differential expression analysis were performed using the ROSALIND cloud-based pipeline (https://rosalind.onramp.bio/). The ROSALIND pipeline generates read distribution percentages, violin plots, identity heatmaps, and sample MDS plots. Normalization, fold change, and significance were calculated using criteria provided by NanoString. ROSALIND follows the nCounter protocol by dividing counts with the geometric mean of the internal normalizer probes within the same lanes. The reference probes selected for normalization are chosen based on the *geNorm* algorithm [70]. Fold changes and *p*-values were calculated using the fast method described in the nCounter Advanced Analysis 2.0 User Manual.

### ATAC-seq

BEAS-2B cells (non-transformed; iAs-transformed; empty plasmid vector; and/or SATB2-expressing cells) were grown to 70–90% confluency, cryopreserved, and sent to ActiveMotif (Carlsbad, CA) for ATAC-seq. Briefly, cell pellets were resuspended in a lysis buffer, and nuclei were pelleted by centrifugation. Genomic DNA was recovered from ~ 1 × 10^5^ nuclei, tagmented using an Illumina Nextera Library Prep Kit, and the resulting DNA fragments purified with a Qiagen MinElute PCR purification kit. Tagged DNA fragments were amplified through 10 PCR cycles, and amplified products were purified with Beckman Coulter Agencourt AMPure SPRI beads. Libraries were quantified using the KAPA Library Quantification Kit and then subjected to paired-end 42 (PE42) sequencing on an Illumina NextSeq 500 sequencer.

Illumina base-call data was processed and demultiplexed with Bcl2fastq2 (v2.20). Adapter sequences were eliminated using skewer [71] v.0.2.2, duplicates were removed with Samtools [72] (v0.1.19), and then the remaining sequences aligned to the hg38 reference genome using the BWA algorithm [73] (mem mode; default settings). Sequencing reads were processed as matched pairs, and only uniquely mapped reads with a mapping quality ≥ 1 were retained for subsequent analysis. To generate genome-wide coverage data, the alignments were extended in silico to a fixed length of 200 bp, and assigned to 32 bp bins along the genome. Bigwig files were created using deeptools [74] v3.5.1.

For comparative analysis of chromatin accessibility, we normalized data by down-sampling the usable number of tags for each sample in a group to the level of the sample in the group with the fewest usable number of tags. Peaks were called using the MACS 2.1.0 algorithm [75] without input control and with the ‘--nomodel’ option, applying a cutoff of *p* ≤ 10^− 7^. Any peaks that appeared in ENCODE false peak blacklists [76, 77] were removed, and a fraction of reads in peaks (FRIP) value ≥ 10% was considered good data quality. Signal maps and peak locations were used as input data for further analysis in the Active Motif proprietary algorithm, which generates sample comparisons for peak metrics, peak locations, and gene annotations. Peak locations were used to annotate ENCODE-predicted cis-regulatory elements (cCRE). Peaks were annotated to the nearest gene using the *annotatePeak* function in ChIPseeker v1.34.1 [78] with the parameters ‘tssRegion = c(-3000, 500)’. We also used GenomicRanges v1.50.2 [79] to annotate peaks with at least a 1 bp overlap with ENCODE3-predicted cis-regulatory elements [76]. Differentially accessible peaks were selected based on specific peak metrics, including an absolute shrunken log2 fold change > 0 and *p*_*adj*_ < 0.05.

Motif enrichment analysis was performed on ATAC-seq peak regions using accessible chromatin sequences as the foreground set. Because ATAC-seq captures accessible chromatin across both promoter-proximal and distal regulatory regions (including enhancers), background sequences were not restricted to promoter regions alone. Enrichment was calculated relative to background genomic regions, and matched for GC content and sequence length to control for nucleotide composition biases (consistent with established motif discovery approaches). Motif analysis was then conducted using the “findMotifsGenome.pl” script in HOMER [80]. During background generation, HOMER selects genomic regions proximal to transcription start sites, matches the GC-content distribution of the query sequences, and weights background regions to account for differences in 1-, 2-, and 3-mer composition relative to the foreground sequences.

For pathway enrichment analysis, a given ATAC peak was removed if the distance to the nearest transcription start site (TSS) was > 100 kb or if it was annotated as ‘distal intergenic’ and did not overlap with the ENCODE cCRE promoter-like (PLS), proximal enhancer-like (pELS), or distal enhancer-like (dELS) regions. Peaks were grouped together if they were annotated to the same nearest gene. A given gene was considered upregulated if there were more significantly upregulated peaks than down, and vice versa for downregulated genes. The resulting gene lists were input into the *compareCluster* function in clusterProfiler v4.6.2 with a default significance cutoff of *p*_*adj*_ ≤ 0.05. We then used msigdbr v7.5.1 to retrieve MSigDB annotations. Dot plots of the enrichment results were created using enrichPlot v1.18.4.

### mRNA stability assay

iAs-transformed and si*circ3915*-KD BEAS-2B cells were seeded at ~ 3–5 × 10⁵ cells per well in 6-well plates and cultured overnight to reach approximately 70–80% confluency. Transcriptional arrest was induced by adding actinomycin D to a final concentration of 5 µg mL^− 1^; control wells received an equivalent volume of DMSO alone. Cells were then harvested at 0, 1, 2, 4, 8, and 24 h after adding actinomycin D. Total RNA was purified and *SATB2* mRNA quantified by RT-qPCR as described above.

### Antisense oligonucleotide (ASO) pulldown

The mature *circ3915* sequence was retrieved from the CircInteractome database (https://circinteractome.nia.nih.gov), and a 30-nucleotide antisense oligonucleotide designed against the *circ3915* backsplice junction (5’ GACAGGAATCATCAAACCTTTAATCTTC); a nonsense oligonucleotide served as the negative control (5’ TTAGTCGACATGTAAACCA). Both ASOs were synthesized by Integrated DNA Technologies with a 3’ Biotin-TEG tag (TEG = 15 atom triethylene glycol linker). Total RNA was purified from ~ 5 × 10^6^ cells and affinity pulldown performed as described by Das et al. [81], with minor modifications. After hybridization, magnetic beads (Dynabeads MyOne Streptavidin C1) were first washed and equilibrated in lysis buffer according to the manufacturer’s instructions. Beads (~ 50 µL per sample) were then added to the ASO–RNA complexes and incubated at 4 °C for 1 h with rotation. Beads were then washed five times with cold lysis buffer, and bead-bound RNA was extracted directly by resuspending the beads in TRIzol reagent.

RNA quality and quantity were assessed using the Agilent small RNA Bioanalyzer chip and Promega QuantiFluor RNA System. Libraries were prepared by the Van Andel Genomics Core from 25.8 ng of isolated small RNA using the Takara SMARTer smRNA-seq kit for Illumina, as per the manufacturer’s protocol. Briefly, the RNA was polyadenylated and then converted to cDNA using Takara’s template switching technology. Samples were PCR amplified for 11 cycles and uniquely indexed with the HT for Illumina primer set. For the final clean-up, we used the recommended nucleoSpin Gel provided with the kit and the optional additional size selection to remove large material incompatible with sequencing. The quality and quantity of the finished libraries were assessed using the Agilent DNA High Sensitivity chip, the Promega QuantiFluor dsDNA System, and Kapa Illumina Library Quantification qPCR assays. Individually indexed libraries were pooled and 75 bp, paired-end sequencing was performed on an Element AVITI sequencer using two Element Biosciences mid-output, 75 bp sequencing kits to an average depth of 15 M reads per sample. Base calling was performed on-instrument, and AVITI OS (v2.6.2) output was demultiplexed and converted to FastQ format with Element Biosciences Bases2fastq v2.20.0.

Normalized counts (from edgeR) were log2-transformed, and RNAs identified in the nonsense control sample subtracted from the ASO samples. Resulting miRNAs were then ranked by robust rank aggregation (RRA) [82]. The RRA score can be treated like a *p*-value, where the null hypothesis is that the two sets of rankings are uncorrelated. We also used the multiMiR R package [83] to identify miRNAs that are either validated or predicted to bind to the *SATB2* mRNA, and generate heatmaps for miRNAs with an RRA score ≤ 0.05.

### Protein extraction

Cells were grown in 75 cm^2^ cell culture flasks (Nest Scientific) and detached in 0.25% Trypsin-EDTA. Approximately 1 × 10^7^ cells were washed with Dulbecco’s PBS, and resuspended in 0.5 mL of radioimmunoprecipitation assay (RIPA) lysis and extraction buffer that was supplemented with 1 mM phenylmethylsulfonyl fluoride, HALT protease and phosphatase inhibitor cocktail, 10 mM 3-methoxybenzylamine (3-MBZ), and 0.5 mM benzylamine (BZA). Cell suspensions were incubated on ice for 30 min, gently vortexed, sonicated for 6 cycles (10 s on/10 sec off) in a Diagenode Bioruptor300, and then centrifuged at 16,000 x g for 10 min at 4 °C. The resulting supernatants were collected in a fresh tube and on ice, and quantified using a Pierce BCA Protein Assay Kit.

### Western blots

Proteins were separated on precast polyacrylamide gels (NuPAGE 4 to 12%, Bis-Tris, 1.0–1.5 mm), and transferred to iBlot 2 Transfer Stacks (Invitrogen) using an iBlot 2 Gel Transfer Device. Membranes were then washed in 25 mL 1x TBS (50 mM Tris-Cl, 150 mM NaCl, pH 7.5) for 5 min at room temperature, and incubated for 1 h at room temperature in 10 mL 1x TBST containing 1–3% BSA. Membranes were then washed three times for 5 min each with 15 mL of TBST + BSA at room temperature, and incubated overnight at 4 °C with gentle agitation in primary antibody mixed to the desired concentration with primary antibody dilution buffer (1x TBST + BSA). Membranes were again washed three times in 15 mL TBST, and then incubated in 10 mL of blocking buffer containing the appropriate HRP-conjugated secondary antibody for 1 h at room temperature. Primary antibodies targeted SATB2 (Invitrogen #PA5-83092; 0.1 mg mL^− 1^; diluted 1:2000); GAPDH (as a loading control; Abcam #ab9485; 0.94 mg mL^− 1^; diluted 1:3000); and anti-HiBiT (Promega #N72000; 1 mg mL^− 1^; diluted 1:1000). An HRP-conjugated IgG anti-mouse or anti-rabbit IgG (Proteintech SA00001-1 or SA00001-2; 0.2 mg mL^− 1^; diluted 1:100000) was used as a secondary antibody for chemiluminescent imaging. For chemiluminescence, we used a SuperSignal West Dura Extended Duration Substrate and the manufacturer’s recommendations (ThermoFisher Scientific) to develop the signal. To detect HiBiT-tagged circ3915p, we used the Nano-Glo HiBiT Blotting system as per the manufacturer’s instructions (Promega). Cancer markers were detected with the EMT Antibody Sampler Kit (Cell Signaling Technologies) according to the manufacturer’s instructions. KRAS pathway markers (MEK, pMEK, ERK, pERK, NFκB) were detected as described above, using primary Cell Signaling Technology and Proteintech antibodies listed in the Key Resources Table. For all blots, developed membranes were placed between transparent plastic sheets or plastic wrap, and images acquired with a ChemiDoc MP imaging system (BioRad).

### IP-MS

Total protein lysates were generated from transfected BEAS-2B cells expressing GFP-tagged *SATB2* and 3×FLAG tagged *circ3915*, as described above. SATB2 and circ3915p were then immunoprecipitated from 1 mg crude protein lysates using the FLAG Immunoprecipitation Kit according to the manufacturer’s protocols (Millipore Sigma). Briefly, lysates were incubated at 4 °C overnight with 40 µL of anti-FLAG M2 monoclonal antibody agarose affinity gel (anti-FLAG M2 affinity gel). The next day, the gel was washed four times with 1x wash buffer and five times with 1 mL of 25 mM ammonium bicarbonate. Agarose beads were then resuspended in 20 µL of sodium bicarbonate for mass spectrometry, and provided to the Michigan State University Integrated Mass Spectrometry Unit, where the ammonium bicarbonate buffer was removed and replaced with acetonitrile. Beads were briefly vortexed and incubated for 1 h at room temperature, condensed with a magnetic bead stand, and the solvent was combined with the buffer. Samples were dried to completion at 30 °C, resuspended in 50 µL of 50% acetonitrile + 25 mM ammonium bicarbonate, and digested overnight at 37 °C with 1 µg of trypsin and 500 ng of LysC. Samples were again dried to completion at 30 °C and resuspended in 50 µL 2% acetonitrile, 0.1% formic acid. Each analysis used 5 µL of sample and a 60 min gradient, followed by one blank cycle. Samples were separated on a C18 EasySpray column (A = 0.1% formic acid, B = 0.1% formic acid in acetonitrile). The mass spectrometry analysis consisted of one full-scan MS followed by top 20 ddMS2. Database searching and protein identification used Proteome Discoverer 2.2.0 via SEQUEST HT against the Uniprot *Homo sapiens* (UP000005460) database.

### IP-MS validation by western blot

Approximately 3 × 10^6^ SATB2-GFP-circ-3×FLAG tagged cells were lysed in Pierce IP Lysis Buffer supplemented with HALT protease/phosphatase inhibitor, sonicated in a Diagenode Bioruptor 300 for 6 cycles of [10s on/10s off], centrifuged at 16,000 × g for 10 min at 4 °C, and 10% of each lysate reserved as the “input” sample for western blots. For the remaining lysate, 50 µL Dynabeads Protein G were first washed twice in cold 1× lysis buffer, and then pre-bound to SATB2/anti-FLAG M2 antibody (3–5 µg) for 20–30 min at 4 °C. Antibody–bead complexes were then washed once in cold lysis buffer and incubated overnight at 4 °C and constant rotation with 200 µg total lysate protein in a final volume of 200 µL IP Lysis buffer plus HALT inhibitors. The next day, beads were collected, and antibody-bound proteins eluted by boiling the beads in 30–35 µL elution buffer (2x Laemmli buffer + 5% β-mercaptoethanol in lysis buffer). The input sample and IP-eluates were then resolved by SDS–PAGE and analyzed by western blot as described above, using commercial antibodies against DDX17, Lamin, SUPT16H, H2A.Z, H2A.X (1H65L16), H2A, H2B, SATB2, and FLAG M2 (as listed in the Key Resources Table).

### Immunocytochemistry

SATB2-GFP-circ-3×FLAG tagged BEAS-2B cells were cultured as described above. Cells (~ 1 × 10^5^) were seeded onto poly-L-lysine, collagen, or gelatin-coated coverslips in 12-well plates (Corning), and incubated overnight at 37 °C. The culture medium was removed, cells gently washed in 1x PBS at room temperature, and fixed in 4% paraformaldehyde for 15 min. Fixed cells were then incubated for 10 min at room temperature in 1x PBS + 0.5% Triton-X 100, washed in 1x PBS, blocked for 1 h in 1x PBS containing 5% goat serum, and then incubated overnight at 4 °C with mouse anti-FLAG M2 antibody (Millipore Sigma F3165; 1 µg mL^− 1^; diluted 1:1000) in 1x PBS containing 5% BSA. The next day, cells were washed three times in 1x PBS and then incubated in the dark for 1 h at room temperature with anti-mouse Alexa Fluor 594 secondary antibody (Invitrogen #A-11005; 2 mg mL^− 1^; diluted 1:1500). Cells were washed 3 times in 1x PBS, stained with DAPI (Sigma Aldrich; diluted 1:1000 in PBS) for 10 min at room temperature, and washed three times in 1x PBS. Coverslips were mounted onto glass slides with ProLong Diamond Antifade Mountant (Invitrogen), and images were captured with a Zeiss 880 confocal microscope and Olympus cellSens software. 

Immunofluorescence experiments were performed on two independent cell preparations.

### Quantification and statistical analyses

Most categorical data were compiled in GraphPad Prism (v10.0), and pairwise comparisons were performed with a Student’s t-test or one-way ANOVA within GraphPad. Statistical details are found in the associated figure legends. Software and methods for analyzing RNA-seq or ATAC-seq data are described in the Methods section above, with software itemized in the Key Resources Table.

De-identified patient data were retrieved from the TCGA database and included 150 entries from lung cancer patients who had a *KRAS* mutation and 342 entries from lung cancer patients who did not have a *KRAS* mutation. We used Kaplan–Meier curves [84] to examine differences in categorical (quantile) *SATB2* expression levels and patient survival, and to detect violations of the proportional hazards assumption. We used differential expression analysis (|log_2_(FC)| > 1, *p* < 0.01) and the Cox Proportional Hazard method to control for continuous *SATB2* expression levels and age at diagnosis, gender, race (filtered for White and Black/African American individuals due to limited data availability for other groups), and smoking status (calculated in pack years). Models were limited to patients without *KRAS* mutations and were censored for either two-year (*N* = 192 without a *KRAS* mutation, 100 with a *KRAS* mutation) or long-term survival (filtering for individuals who survived at least two years, and tracking them until the last follow-up date, *N* = 53 without a *KRAS* mutation, 16 with a *KRAS* mutation). We then used Cox proportional hazard models [85] to assess the univariate prognostic significance of tumor variables on overall survival. Hazard ratios and *p*-values were obtained from these models, where *p*-values < 0.05 were considered to indicate statistical significance.

## Results

### Inorganic arsenic dysregulates oncogenic pathways

It is well known that chronic, low-dose iAs exposure (e.g., via drinking water) causes lung cancer [86]. We therefore exposed BEAS-2B human bronchial epithelial cells to 2 µM iAs for 12 weeks, an established low-dose (but rapid) exposure model that faithfully recapitulates the epithelial to mesenchymal transition (EMT) [25].

**Fig. 1:**
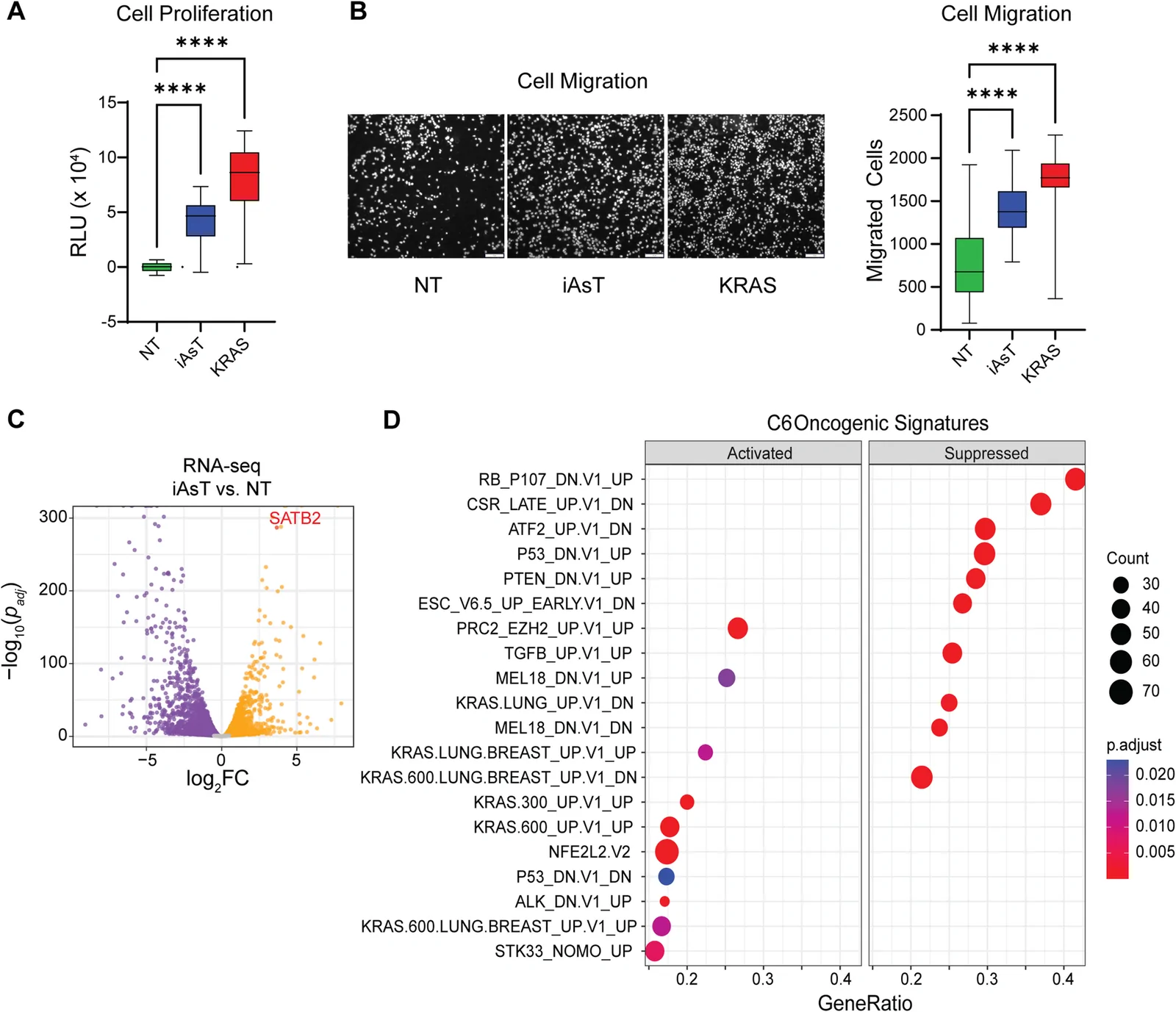
Inorganic arsenic (iAs) promotes cell transformation by driving oncogenic gene expression programs. Data were generated from the rapid (12 week) exposure model. **A**, Proliferation of non-treated (NT), iAs-transformed (iAsT), and KRAS/p53 mutant BEAS-2B cells 72 h after seeding. Luminescence was measured with a CellTiter-Glo ATP assay. Values were normalized to background and calculated relative to NT cells. *N* = 3 independent experiments with 6 technical replicates each. RLU = relative light units; **** *p* < 0.0001. **B**, Representative images (top) of migrating NT, iAsT, and *KRAS/p53* mutant BEAS-2B cells after 24 h in a transwell assay. Cells were fixed and stained with DAPI, and cell counts were obtained using an Olympus cellSens counting module. Cell numbers (bottom) were normalized to NT control cells. *N* = 3 independent experiments with 3 technical replicates each; ****p < 0.0001. Scale bars = 100 μm. **C**, Differentially expressed genes (DEGs) in iAs-transformed BEAS-2B cells. Purple dots represent downregulated genes with a *p*_*adj*_ <0.05, FC <-1.5; orange dots represent upregulated genes with a *p*_*adj*_ < 0.05, FC > 1.5. Grey dots represent genes with FC < 1.5, > -1.5. In total, there were 3517 upregulated and 4625 downregulated genes. **D**, Top enriched MSigDB C6 oncogenic signatures in the iAsT DEGs

BEAS-2B cells harboring an activating *KRAS* G12V mutation were used as a positive control for lung cell transformation and were a generous gift from the Brainson lab [87]. As expected [25, 61], and consistent with other reports [88, 89], chronic 2 µM iAs exposure transformed BEAS-2B cells into a more proliferative and migratory state (Fig. 1A, B). We identified 8142 differentially expressed genes in iAs-transformed cells (DEGs; Fig. 1C and Fig. S2A-B) and validated selected genes using the NanoString nCounter system (Fig. S2C). Some of the most dysregulated genes (Table S5, Fig S2B) included oncogenes like *MMP1* [90] and the *GAS1* tumor suppressor [91], and oxidative stress response genes like *HMOX1* [92]. We were particularly intrigued by *SATB2* upregulation, since SATB2 is a master regulator of chromatin accessibility [93] and *SATB2* over-expression is associated with certain cancers [47]. Molecular signatures database (MSigDB), KEGG, and Reactome pathway analysis also showed dysregulated oncogenic pathways, including cell-substrate junction organization, axonogenesis, Wnt signaling, Hippo signaling, TGF-β, NCAM1 interactions, and KRAS signaling (Fig. S2D-G). *KRAS* dysregulation was also identified in a C6 oncogenic signature analysis (Fig. 1D). Thus, iAs induces a KRAS-like transcriptional state in transformed lung epithelial cells.

**Fig. 2:**
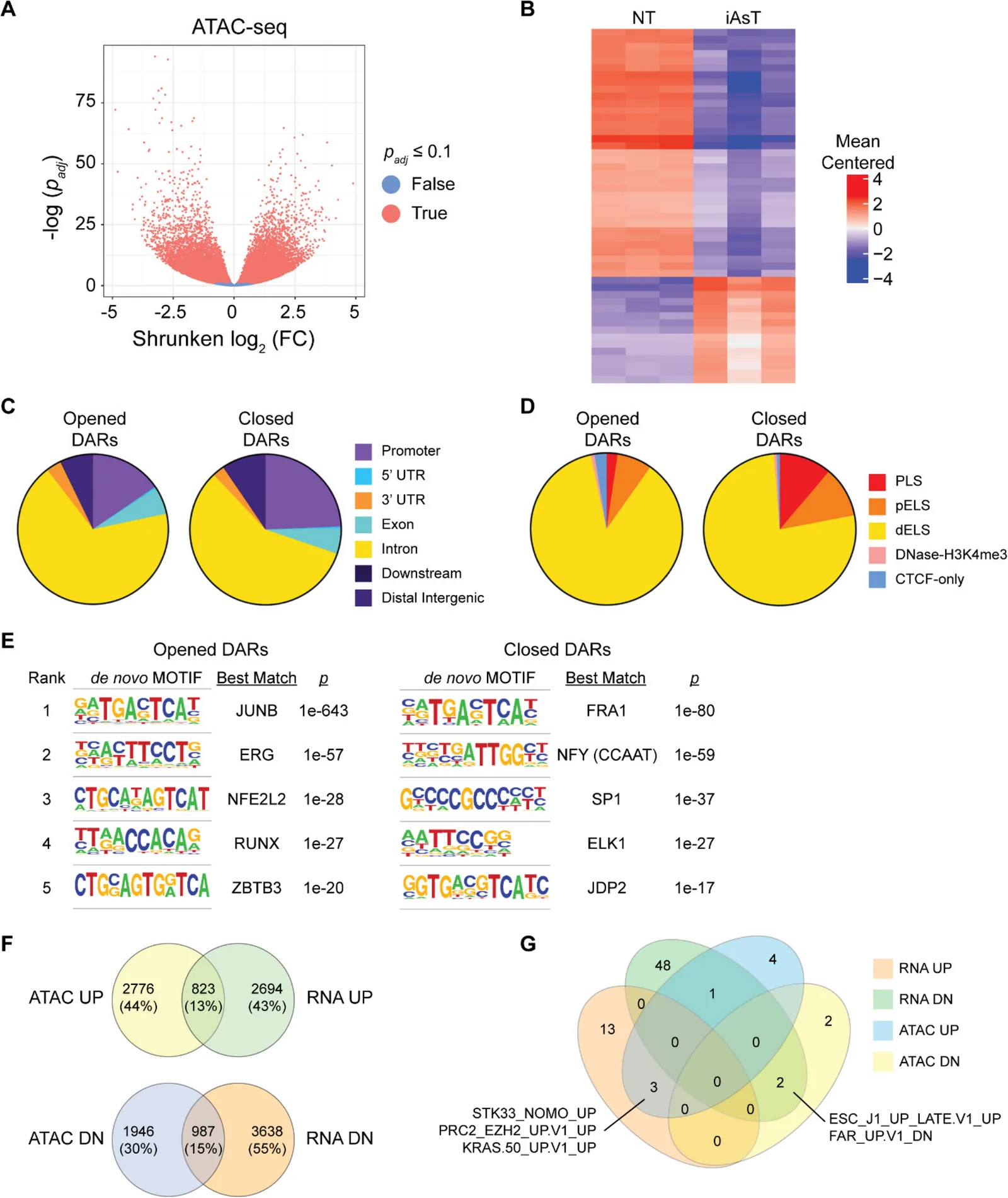
Inorganic arsenic (iAs) reorganizes chromatin structure around known oncogenes/pathways. iAs-transformed cells were generated with the rapid (12-week) transformation model. **A**, Volcano plot of differentially accessible regions (DARs) by ATAC seq between non-transformed (NT) and iAs-transformed (iAsT) BEAS-2B cells. DARs were defined by a *p*_*adj*_ ≤ 0.1. In total, there were 19,531 “closed” DARs and 19,875 “opened” DARs. **B**, Heatmap of the top 50 DARs between non-transformed (NT) and iAs-transformed (iAsT) cells. Rows are the top genes; columns are the mean centered peak values for each gene in each sample. **C**, Genomic distribution of all DARs in NT and iAsT cells. DARs regions were defined by *p*_*adj*_ < 0.01 and Shrunken Log_2_(FC) > 1 for opened DARs, and ShrunkenLog_2_(FC) < -1 for closed DARs. Of the 12,063 DARs that met these criteria, 6753 were in opened chromatin and 5310 were in closed chromatin. **D**, Proportion of DARs in NT and iAsT cells that are annotated as ENCODE candidate Cis-Regulatory Elements (cCREs). PLS = promoter-like signatures; pELS = proximal enhancer-like signatures; dELS = distal enhancer-like sequences. In total, there were 10,123 DARs (82.4%) that mapped to ENCODE cCREs, of which 5790 were in opened chromatin regions and 4333 were in closed chromatin regions. **E**, Top enriched *de novo* transcription factor motifs and best matching transcription factors in iAsT opened and closed DARs. **F**, Congruence between iAsT DARs and proximal gene expression. ATAC UP = opened chromatin; ATAC DN = closed chromatin; RNA UP = upregulated transcripts; RNA DN = downregulated transcripts. Counts are the number of individual genes above the minimum fold-change threshold for mRNA expression (> 1 Log_2_(FC)) or open chromatin (> 1 ShrunkenLog_2_(FC)). **G**, MSigDB oncogenic signatures in iAs-transformed cells that were commonly enriched in DARs and DEGs. ATAC UP = opened chromatin; ATAC DN = closed chromatin; RNA UP = upregulated transcripts; RNA DN = downregulated transcripts

Since *SATB2* (a chromatin organizer) was among the most dysregulated genes in iAs-transformed cells, we focused on SATB2 as a candidate regulator of the KRAS-like transcriptional changes. We used ATAC-seq to identify differentially accessible regions (DARs) and determine if transcriptional changes were accompanied by alterations in chromatin accessibility. We identified ~ 34,000 differentially accessible regions (DARs) (Fig. 2A; Table S6; Fig. S3A, B), distributed across promoters and gene bodies (Fig. S3C). We also demonstrate congruence between genes associated with open and closed chromatin in iAsT cells (Fig. S3D).

Hierarchical clustering of the top 50 DARs revealed distinct accessibility patterns between NT and iAsT cells (Fig. 2B). We also identified 12,063 high-confidence DARs, of which 6,753 were in opened and 5,310 were in closed chromatin regions, distributed broadly across the genome (Fig. 2C). DARs were enriched at regulatory elements, with increased accessibility at ENCODE distal enhancer-like signatures and decreased accessibility at promoter-like and proximal enhancer-like regions (Fig. 2D). Association of DARs with nearby genes (≤ 1 kb) revealed enrichment of pathways related to cell adhesion, ERBB signaling, Hippo signaling, Wnt signaling, PI3K/AKT signaling, the epithelial-to-mesenchymal transition (EMT), and KRAS/EGFR signaling (Fig. S3E–I). Motif analysis further showed that regions with increased accessibility were enriched for transcription factor binding sites associated with KRAS-related signaling (e.g., JUNB and ERG), as well as NFE2L2, RUNX, and ZBTB3. In contrast, regions with decreased accessibility were enriched for motifs corresponding to FRA1, NFY, SP1, ELK1, JDP2, and members of the CCAAT/enhancer binding protein (C/EBP) family (Fig. 2E; Table S7). Integrating chromatin accessibility and gene expression data revealed partial concordance between DARs and transcriptional changes (Fig. 2F). Specifically, regions with increased accessibility were enriched near upregulated genes in oncogenic gene sets such as STK33_NOMO_UP, PRC2_EZH2_UP.V1_UP, and KRAS.50_UP.V1_UP, whereas regions with decreased accessibility were enriched near downregulated genes in ESC_J1_UP_LATE.V1_UP and RAF_UP.V1_DN gene sets (Fig. 2G). Together, these findings indicate that iAs exposure is associated with widespread reorganization of chromatin accessibility at regulatory loci, including regions linked to KRAS- and NFE2L2-related transcriptional programs. Our ATAC-seq analysis demonstrates that SATB2 expression is associated with altered chromatin accessibility at these regions; however, in the absence of SATB2 ChIP-seq data, we cannot determine whether these effects reflect direct SATB2 occupancy at the affected loci.

### *SATB2* generates a unique circular RNA transcript

**Fig. 3:**
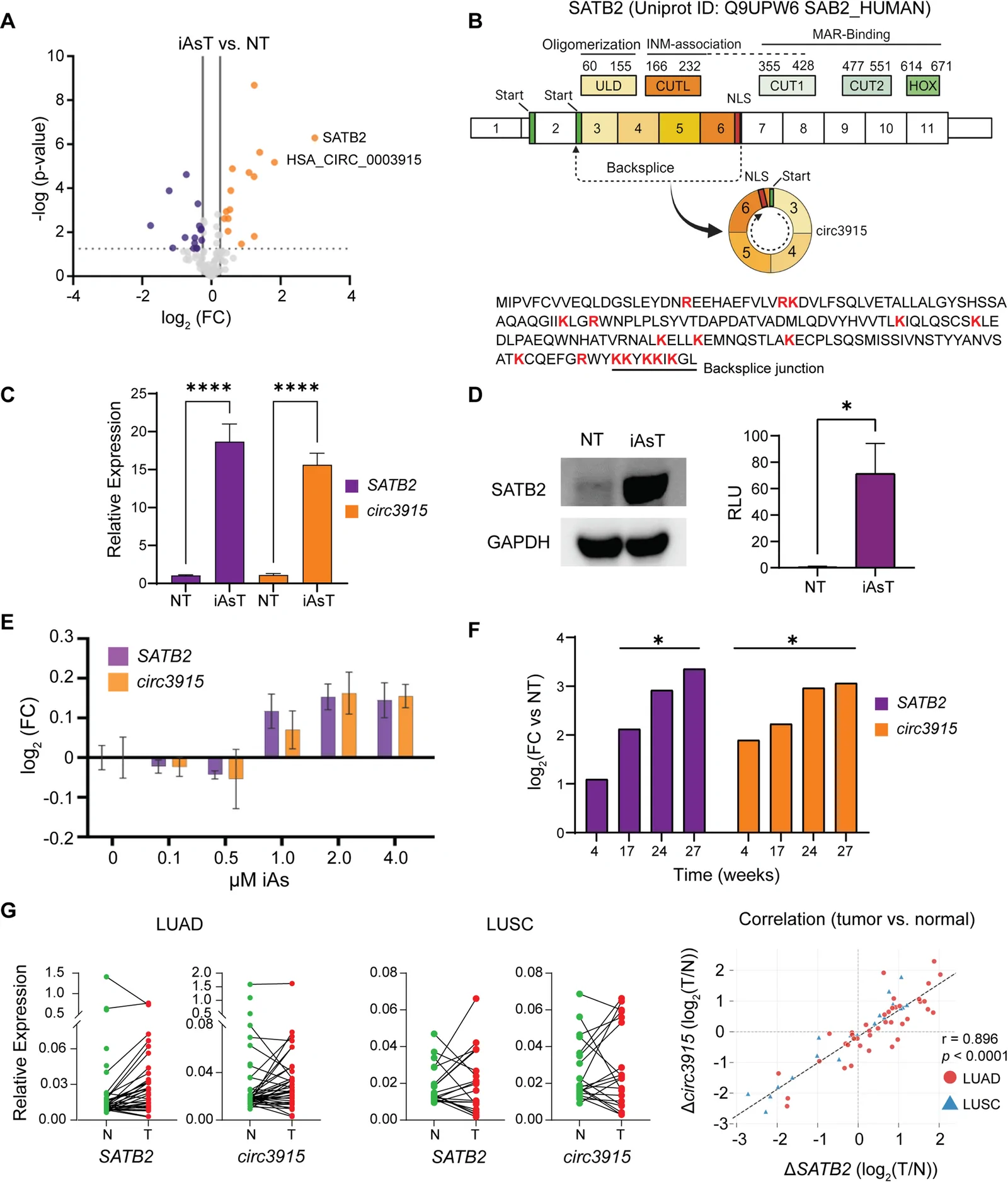
*SATB2* and *circ3915* are upregulated in iAs-transformed cells and lung cancer tumors. Data were generated in the rapid (12 week) iAs transformation model. **A**, NanoString-based detection of differential RNA and circRNA expression in iAs-transformed (iAsT) BEAS-2B cells relative to non-transformed (NT) cells. Upregulated RNAs (orange dots) were defined by *p* < 0.05 and fold change (FC) > 1.2. Downregulated circRNAs (purple dots) were defined by *p* < 0.05 and FC < -1.2. Grey dots represent RNAs with no significant difference in RNA expression. **B**, *SATB2* and *circ3915* transcripts, corresponding peptide domains, and predicted amino acid sequence for circ3915p. ULD = ubiquitin-like domain; CUTL = cut-like domain; CUT1 = cut domain 1; CUT2 = cut domain 2; HOX = homeobox; INM = inner nuclear membrane; MAR = matrix associated region; NLS = nuclear localization signal. CUT domains are specific DNA-binding motifs. The backsplice junction is underlined, and red amino acids are predicted trypsin cleavage sites. **C**, Relative *SATB2* and *circ3915* expression, as measured by RT-qPCR. *SATB2* expression increased 18.7-fold and *circ3915* expression increased by 15.6-fold. *N* = 3 replicates; **** *p* < 0.0001. **D**, Representative western blot and GAPDH-normalized expression showing SATB2 upregulation in iAsT BEAS-2B cells. RLU = relative light units; * *p* < 0.05. **E**, *SATB2* (purple) and *circ3915* (orange) expression in BEAS-2B cells after acute, 48 h exposure to iAs. Fold-change was measured by RT-qPCR and calculated relative to non-treated BEAS-2B cells. **F**, *SATB2* and *circ3915* expression in BEAS-2B cells at different time-points in the two-hit iAs transformation model, as measured by NanoString. *N* = 3 replicates; * *p* < 0.05. **G**, *SATB2* and *circ3915* expression in normal (N) and adjacent tumor (T) samples, as measured by RT-qPCR. All expression data were normalized to the expression of the geometric mean of three common housekeeping genes (*GAPDH*,* TBP*, and *RPII*). The scatter plot shows the association between changes in *SATB2* and *circ3915* (y-axis) expression for LUAD (*n* = 40) and LUSC (*n* = 20) samples, respectively. The dashed line is the linear regression fit across all samples. The Pearson correlation coefficient and two-tailed *p*-value are indicated

iAs exposure can lead to alternative mRNA splicing [25], and circular RNAs are one possible product of alternate mRNA splicing that can regulate cell differentiation [94, 95]. We therefore used the Arraystar Human Circular RNA Array to screen iAs-transformed cells for differentially expressed circRNAs (Fig. S4A, B), and discovered several dysregulated, cancer-associated circRNAs (e.g., hsa_circ_0002490, hsa_circ_0002874, hsa_circ_0075320, and hsa_circ_0008928) [48, 96–98]. Since circRNA microarray data are subject to false-positive signal [99, 100], we also used a custom NanoString analysis to verify the most dysregulated circRNAs identified by microarray. One of the most up regulated circRNAs from both experiments was *circ3915* (Fig. 3A; Fig. S4A), an uncharacterized circRNA that encompasses *SATB2* exons 3–6 (Fig. 3B). *SATB2* mRNA and *circ3915* were co-expressed during iAs-induced transformation (Fig. 3C), with increased *SATB2* expression correlating with increased SATB2 protein production (Fig. 3D). Both transcripts are produced in an iAs dose- (Fig. 3E) and time-dependent manner (Fig. 3F), beginning with the initial iAs exposure and persisting throughout the epithelial-to-mesenchymal transition (EMT). These findings were also replicated in iAs-exposed 16HBE cells (Fig. S4C, D). Head-to-tail splicing of circ3915 was confirmed by PCR amplification with divergent primers and Sanger sequencing, validating the presence of the backsplice junction between exons 3 and 6 (Fig. S4E).

We next investigated if *SATB2* and *circ3915* expression were detectable in human samples. We obtained paired primary tumors and normal adjacent tissues from lung adenocarcinoma (LUAD) and lung squamous cell carcinoma (LUSC) patients. Among the 40 paired LUAD and 20 paired LUSC samples, 24 and eight showed concurrent upregulation of *SATB2* and *circ3915*, respectively (Fig. 3G). Conversely, when *SATB2* expression decreased, so too did *circ3915* expression in seven LUAD and seven LUSC pairs. Thus, *SATB2* and *circ3915* were co-regulated in 31/40 (78%) and 15/20 (75%) of LUAD and LUSC samples, respectively (*r* = 0.896; *p* < 0.0001). Collectively, these data indicate that iAs exposure triggers *SATB2* overexpression in multiple cell lines and exposure models, leading to a novel *circ3915* RNA. Their expression seems to be correlated in human normal and lung tumor specimens (Fig. 3G, right panel), and both RNA species persist through the EMT.

### *circ3915* can be translated, and can generate a circ3915 peptide (circ3915p) that associates with SATB2

**Fig. 4:**
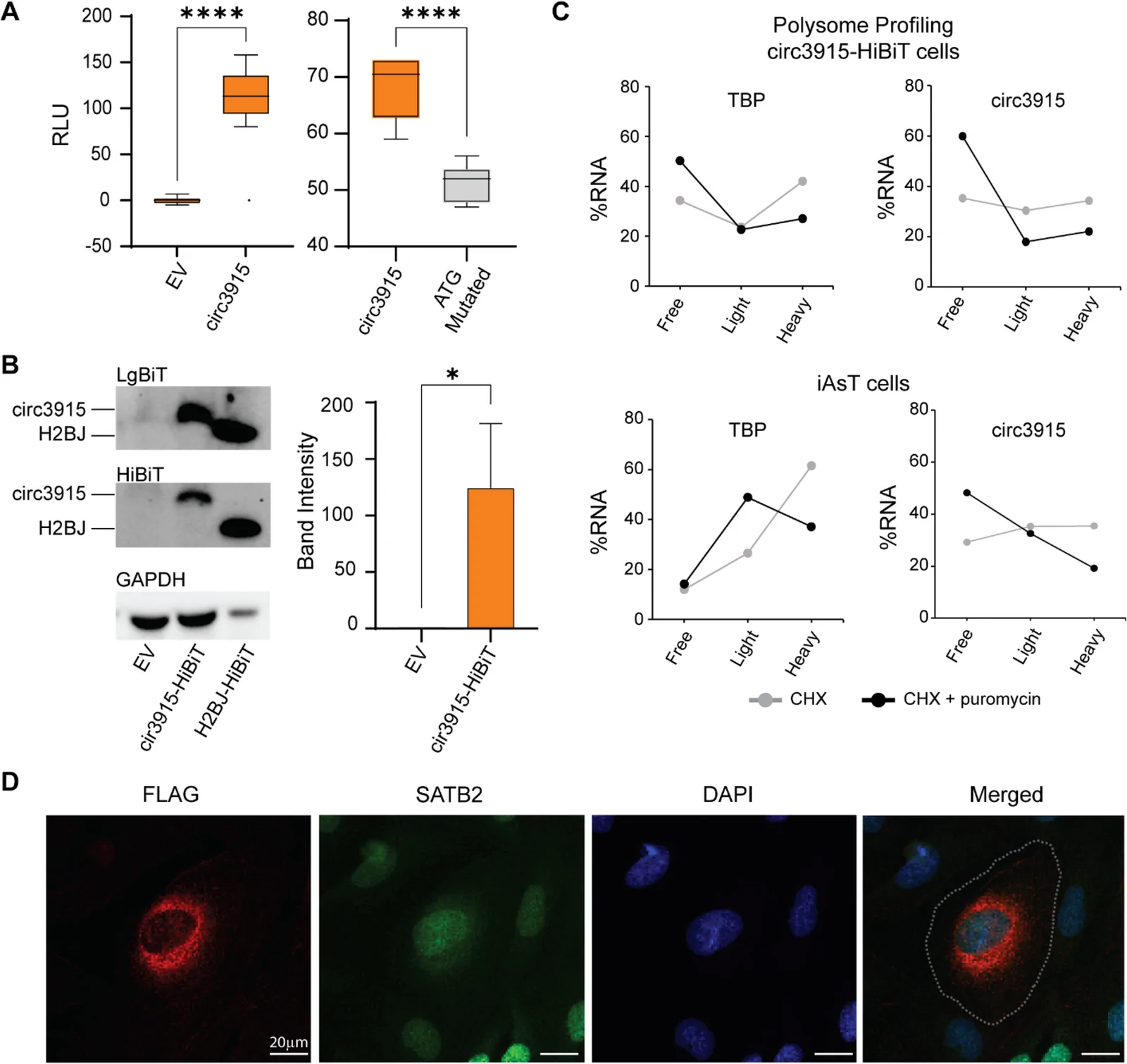
Tagged *circ3915* can be translated, and the derived peptide can associate with SATB2-containing protein complexes. **A**, Bioluminescence in BEAS-2B cells containing an empty vector (EV), cells that are expressing a HiBiT-tagged *circ3915* construct (circ3915), or cells expressing an ATG to GTG mutated *circ3915* construct as measured with a split luciferase translation assay. RLU = relative light units; *N* = 3; **** *p* < 0.0001. **B**, Western blots for 100 µg of total protein or 5 µg of nuclear extract from BEAS-2B cells transfected with an empty vector (EV), HiBiT-tagged *circ3915* construct (circ3915-HiBit), or a HiBiT-tagged histone H2BJ (H2BJ) positive control. HiBiT was detected with either LgBiT (top panel) or an anti-HiBiT antibody (middle panel); * *p* < 0.05. **C**, Endogenous *circ3915* is associated with actively translating polysomes. BEAS-2B cells were either transfected with a circ3915-HiBiT plasmid (top) or transformed with iAs in the rapid (12 week) transformation model (bottom). Line graphs show the distribution of cytoplasmic RNA across sucrose gradient density fractions. Grey lines show the distribution of RNA under normal culture conditions (CHX = cycloheximide), and black lines show the distribution of RNA after adding puromycin (puromycin disrupts translation elongation). An increase in %RNA within the light fractions after treating cells with puromycin indicates that the RNA was being translated under normal conditions. TBP = TATA-box binding protein mRNA; circ3915 = *circ3915* RNA. **D**, Confocal images of BEAS-2B cell nuclei that were transduced with SATB2-GFP and circ3915-HiBiT expression vectors. The dotted line encompasses a single cell and marks the approximate cell membrane boundary. Scale bar = 20 μm

*circ3915* has an in-frame ATG start codon and SATB2 putative nuclear localization signal, oligomerization domain, and inner nuclear membrane domain, but no DNA binding domain (Fig. 3B). We therefore asked if *circ3915* can be translated into a peptide (circ3915p), and if that peptide might have a biological function. Unfortunately, MS/MS methods were unable to detect any fragments containing the circ3915p backsplice junction because of the many lysine residues in the backsplice junction (Fig. 3B), which is a common occurrence in circRNA studies. There is also no commercially available antibody for circ3915p that would not also cross-react with endogenous SATB2. We therefore introduced a tag (HiBiT/3×FLAG) into the *circ3915* expression construct immediately downstream of the backsplice junction and before the start codon (Fig. S5A), and monitored translation by bioluminescence, polysome fractionation, western blot, and mass spectrometry. When we transduced BEAS-2B cells with HiBiT-tagged *circ3915* expression constructs, we saw enhanced in vitro bioluminescence (Fig. 4A), HiBiT protein in western blots (Fig. 4B), and the FLAG tag in mass spectra (Fig. S5B), indicating that circ3915-HiBiT is translated in this experimental system.

Approximately 60% of all endogenous *circ3915* transcripts were associated with actively translated polysomes, with puromycin treatment shifting *circ3915* transcripts towards light and free polysome fractions (Fig. 4C; Fig. S5C); similar results were obtained in iAs-transformed BEAS-2B cells (Fig. 4C). In contrast, an untranslated endogenous SATB2 circRNA (hsa_circ_0008928 [101]) did not associate with polysomes or show a shift in polysome association (Fig. S5D).

Immunofluorescence experiments showed that circ3915 can be translated, and that a tagged *circ3915*-derived peptide (circ3915p) produces a detectable signal in stably transduced BEAS-2B cells (Fig. 4D). We observed partial overlap with SATB2 signal, and AlphaFold3 predictions suggest that circ3915p may interact with SATB2 (Fig. S5E–H). However, the precise circ3915p subcellular localization (e.g., to the nucleus or chromatin) or interactions with SATB2 could not be determined from these images. To test for potential protein-protein interactions, then, we expressed 3×FLAG-tagged circ3915p in non-transformed BEAS-2B cells, immunoprecipitated FLAG-tagged protein complexes, and analyzed associated proteins by mass spectrometry. After filtering out keratins and proteins detected in empty vector controls, we identified protein sets unique to SATB2, unique to circ3915p, and shared between both (Table S8). Notably, SATB2 peptides (69% coverage) were detected in samples expressing circ3915p, indicating that a circ3915-derived peptide can be detected in association with SATB2-containing protein complexes in total cell lysates. However, because these experiments used tagged constructs and whole-cell lysates, they do not establish compartment-specific interactions or direct nuclear binding.

Interestingly, SATB2 and circ3915p also immunoprecipitated with chromatin and chromatin remodelers (H1, H2A.Z, macroH2A, and HDAC2), splicing factors (SNRPB, SNRPD2, SNRPD3, SRSF3, SRSF6, SRSF9, TRA2B), and the nuclear envelope protein BANF1 (Table S8). IP-MS data were validated by western blots (Fig. S5I-J), showing that SATB2 immunoprecipitants were enriched for chromatin remodelers and nuclear proteins such as DDX17, Lamin, SUPT16H, H2A.Z, H2A.X, H2A, and H2B. In contrast, immunoprecipitating the circ3915p-3×FLAG tag pulled down SATB2 but not histones, consistent with a model where circ3915p indirectly associates with repressive complexes via SATB2, rather than directly binding chromatin.

The chromatin proteins associated with both SATB2 and circ3915p are usually associated with transcriptional repression, while chromatin proteins unique to SATB2 (e.g., H2A.X, PARP1, and SSRP1) are often associated with active chromatin. These results suggest SATB2 regulates gene expression by directing changes to chromatin structure. HDAC2 association with circ3915p further suggests that circ3915p (which lacks SATB2 DNA binding domains; Fig. 3B) might sequester repressive complexes away from cis-regulatory elements. SATB2 and circ3915p both interact with CEBPB, which is consistent with the enriched transcription factor motifs in differentially accessible genomic regions (below). Taken together, these data indicate that *circ3915* can be translated, and that circ3915p can associate with SATB2 under experimental conditions (presumably to modify chromatin architecture).

### *circ3915* stabilizes the linear *SATB2* transcript

**Fig. 5:**
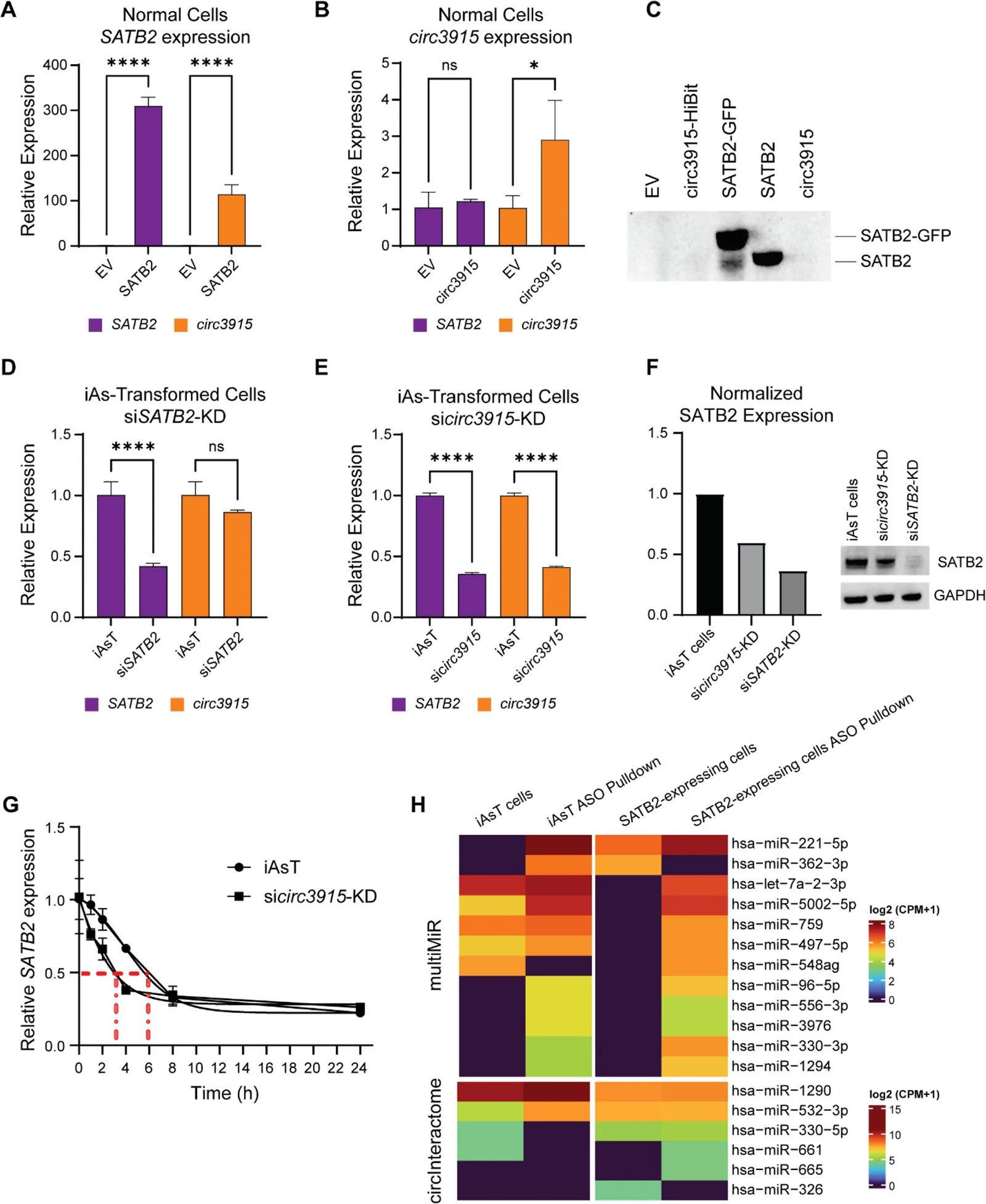
*circ3915* protects the linear SATB2 transcript. **A**, Relative *SATB2* (purple) and *circ3915* (orange) expression in BEAS-2B cells containing an empty vector (EV) or *SATB2* expression vector (SATB2), as measured by RT-qPCR. *N* = 3 replicates; **** *p* < 0.0001. **B**, Same as **A**, except with a *circ3915* expression vector (circ3915). *N* = 3 replicates; ns = not significantly different; * *p* < 0.05. **C**, Western blot showing ectopic SATB2 and circ3915 expression in non-transformed BEAS-2B cells. Expressing *circ3915* alone does not increase SATB2 levels. **D**, BEAS-2B cells were transformed with iAs (rapid transformation model; iAsT), and then treated with siRNAs to knock down *SATB2* (si*SATB2*-KD). *SATB2* and *circ3915* RNAs were then measured by RT-qPCR. *N* = 3 replicates. ns = not significantly different; **** *p* < 0.0001. **E**, Same as panel **D**, except cells were treated with siRNAs to knock down *circ3915*. *N* = 3 replicates; **** *p* < 0.0001. **F**, Western blot and GAPDH-normalized SATB2 expression in iAs-transformed BEAS-2B cells after knocking down *circ3915* or *SATB2* with target-specific siRNAs. **G**, *circ3915* knockdown reduces *SATB2* mRNA stability in iAs-transformed BEAS-2B cells treated with actinomycin D. *N* = 3 replicates per time point and treatment. Red lines indicate mRNA half-life. **H**, High-confidence, mature miRNAs that interact with *circ3915*

Some circRNAs protect their cognate mRNA from post-transcriptional regulatory pathways [53, 102], and the co-expression of *SATB2* and *circ3915* RNA suggested a similar mechanism. We therefore ectopically expressed *SATB2* or *circ3915* in non-transformed BEAS-2B cells (where *SATB2* mRNA is normally absent) and quantified the corresponding RNAs by qRT-PCR. Ectopic *SATB2* expression increased *SATB2* mRNA and protein levels (as expected) and increased *circ3915* expression. In contrast, ectopic *circ3915* expression had no effect on endogenous *SATB2* mRNA expression or protein levels (Fig. 5A-C). We then used siRNAs to knock down *SATB2* or *circ3915* RNAs in iAs-transformed cells. In this case, knocking down linear *SATB2* decreased *SATB2* mRNA levels (as expected), but had no effect on *circ3915* RNA levels (Fig. 5D). However, knocking down *circ3915* depleted *circ3915* and *SATB2* RNA in iAs-transformed cells (Fig. 5E), with a corresponding decrease in SATB2 protein (Fig. 5F). Thus, whenever there is *SATB2* expression, *circ3915* is generated.

We also treated iAs-transformed BEAS-2B cells with actinomycin D to block transcription and measured *SATB2* mRNA levels over time. *SATB2* mRNA persisted for ~ 6 h in iAs-transformed cells but only persisted for ~ 3 h in *circ3915* knockdown cells (Fig. 5G). We then used antisense oligonucleotide (ASO) methods [81] to pull down and identify candidate miRNAs that may interact with *circ3915*, and used those miRNA sequences, multiMiR, and CircInteractome [103] databases to identify mature miRNAs (Fig. 5H). At least one of these high-confidence miRNAs (miR-330-5p) also interacts with *circSATB2* to regulate cell proliferation, migration, and invasion in lung cancer [49]. Collectively, these data suggest that *SATB2* expression is regulated by *circ3915*, potentially through post-transcriptional mechanisms that may include interactions with miRNAs [52] that would otherwise target *SATB2* mRNA transcripts for degradation or disrupt *SATB2* translation, hypotheses that can be tested in future studies. The data also suggest that *SATB2* and *circ3915* are required to maintain an oncogenic or cancerous cell state.

### SATB2 and *circ3915* epigenetically regulate oncogenic gene expression programs

To determine if SATB2 and *circ3915* are required for cell transformation, we used lentivirus vectors to consistently express *SATB2* mRNA and/or *circ3915* in non-transformed BEAS-2B cells.

**Fig. 6:**
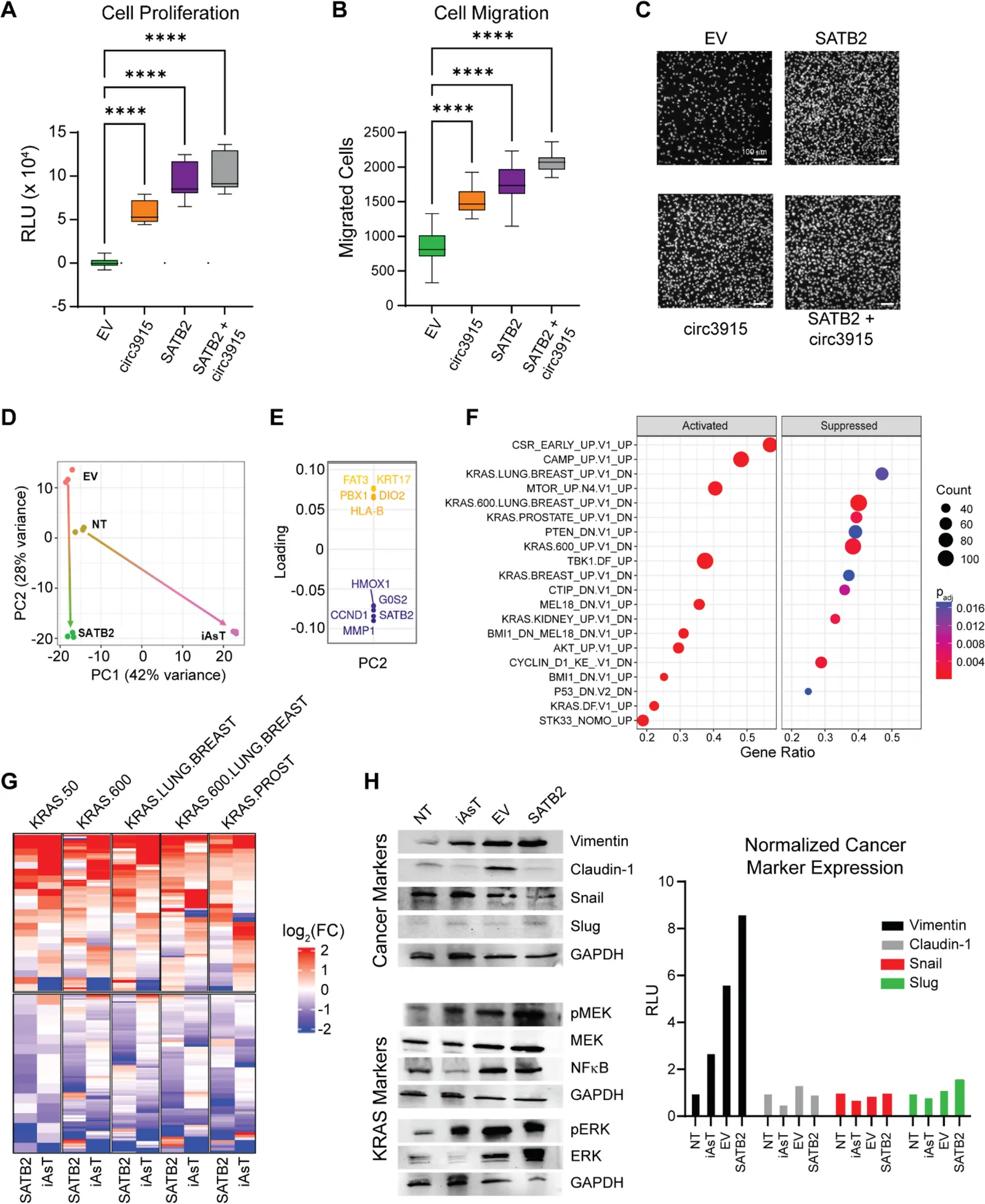
The *SATB2*/*circ3915* axis promotes oncogenic phenotypes and transcriptional reprogramming. **A**, BEAS-2B cell proliferation following ectopic expression of SATB2, circ3915, or both, as measured with a CellTiter-Glo ATP assay 72 h after seeding. EV = non-transformed cells containing an empty lentivirus vector. SATB2 + circ3915 = cells that contained SATB2 and circ3915 expression vectors, and the most pronounced effects were observed when both transcripts were expressed. RLU = relative light units. Luminescence was normalized to background, and values were calculated relative to the control cells. *N* = 3 independent experiments with 6 technical replicates each; **** *p* < 0.0001. **B**, BEAS-2B cell migration after transfection with lentivirus expression vectors, as measured by a transwell migration assay 24 h after seeding. Cells were fixed and stained with DAPI, and cell counts were obtained with an Olympus cellSens counting module. Counts were normalized to the EV cells. *N* = 3 independent experiments with 3 technical replicates each; **** *p* < 0.0001. **C**, Representative images from the transwell migration assay. Scale bars = 100 μm. Note that *circ3915* expression alone was sufficient to increase proliferation and migration in non-treated BEAS-2B cells, despite the absence of detectable endogenous *SATB2* expression. These data suggest that *circ3915* may exert both SATB2-dependent and SATB2-independent effects. **D**, Principal component analysis of RNA-seq expression profiles from non-transformed BEAS-2B cells (NT, brown); non-transformed cells transfected with an empty vector (EV, red); non-transformed cells containing a SATB2 expression vector (SATB2, green); and iAs-transformed BEAS-2B cells from the rapid (12 week) transformation model (iAsT, pink). **E**, The top genes contributing to the variance between PC1 and PC2 in panel **D**. **F**, Top enriched MSigDB oncogenic signatures reflected in the DEGs from non-transformed BEAS-2B cells with ectopic *SATB2* expression. **G**, Relative RNA expression of MSigDB oncogenic KRAS signature genes in non-transformed BEAS-2B cells that express *SATB2* (SATB2) or in iAs-transformed BEAS-2B cells (iAsT, rapid transformation model). **H**, Western blot and GAPDH-normalized cancer and KRAS biomarkers in non-treated (NT) and iAs-transformed (iAsT) BEAS-2B cells, and in BEAS-2B cells containing an empty vector (EV) or a *SATB2* expression vector (SATB2)

Under all conditions, we observed enhanced cell proliferation and migratory potential relative to BEAS-2B cells transduced with an empty lentivirus vector, with the most pronounced effects observed in cells expressing circular and linear transcripts (Fig. 6A-C). *SATB2* dysregulated many genes in non-transformed cells (Fig. S6A-C; Table S9), resulting in a gene expression pattern more similar to iAs-transformed cells than to non-treated cells (Fig. 6D). In fact, expressing *SATB2* in BEAS-2B cells recapitulated some of the gene expression patterns found in iAs-transformed cells (Fig S6D). As in iAs-transformed cells, *SATB2* expression increased *HMOX1*, *GOS2*, *CCND1*, and *MMP1* expression, and decreased *FAT3*, *KRT17*, *DIO2*, *PBX1*, and *HLA-B* expression (Fig. 6E). *SATB2* expression also dysregulated key oncogenic signatures, including a striking enrichment in oncogenic KRAS-like signatures (Fig. 6F-H). Thus, SATB2 and its co-induced *circ3915* epigenetically regulate oncogenic gene expression programs and oncogenic phenotypes, even without iAs exposure or engineered driver mutations.

### SATB2-associated chromatin modifications are associated with KRAS-like gene expression patterns

**Fig. 7:**
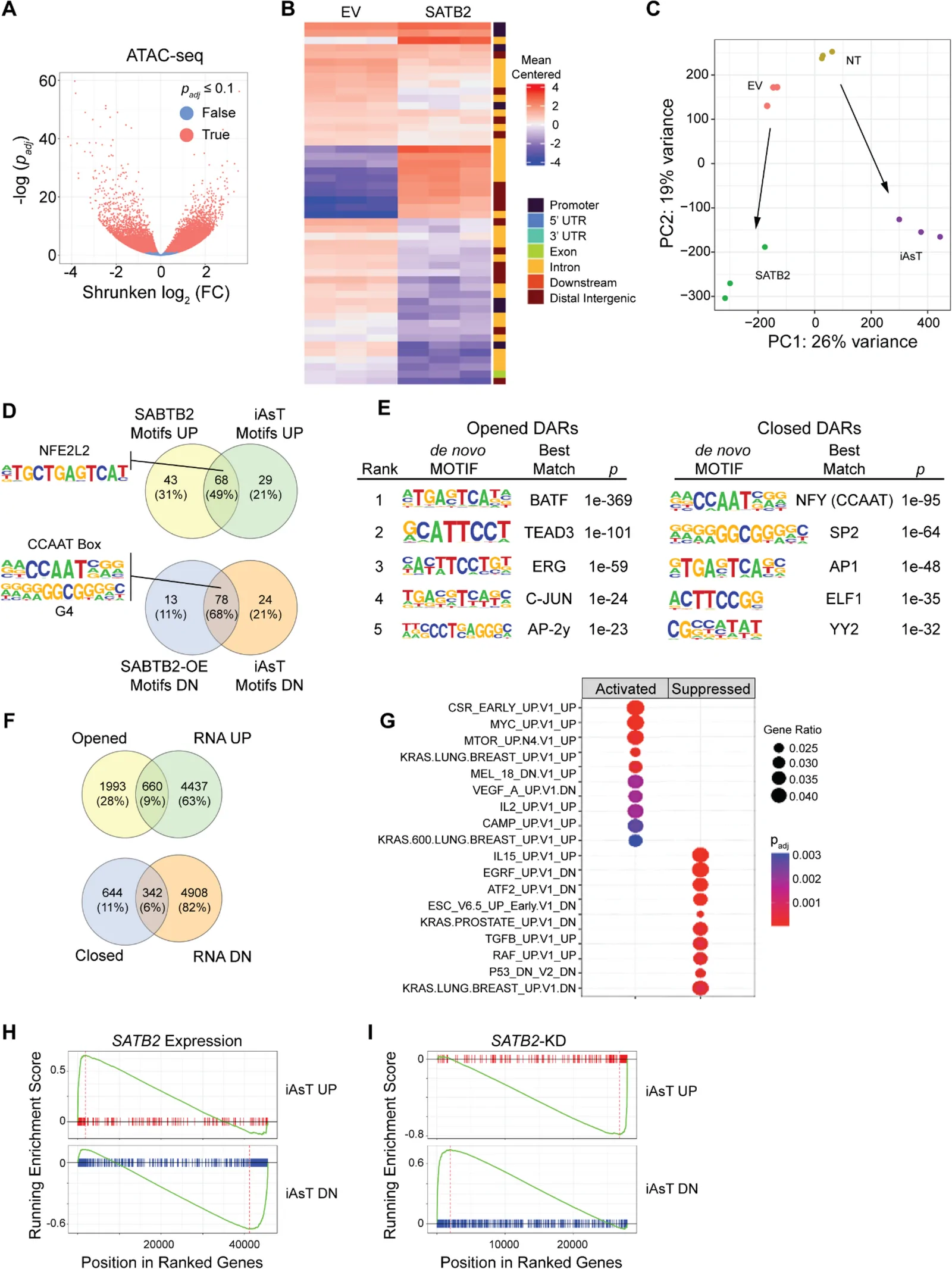
*SATB2* expression alters chromatin accessibility at cis-regulatory elements. **A**, Differentially accessible regions (DARs) between non-treated BEAS-2B cells containing an empty expression vector and BEAS-2B cells containing a SATB2 expression construct. DARs were defined by an FDR (*p*_*adj*_) ≤ 0.1. Of the 99,743 merged peaks, there were 33,397 DARs. SATB2 expression generated 19,383 opened and 14,014 closed peaks. **B**, Heatmap of the top 50 DARs between non-transformed BEAS-2B cells containing an empty expression vector (EV) or expressing *SATB2* (SATB2). Columns are the mean centered peak values for each gene (row) in each sample. The genomic location of each peak is shown to the right of the heatmap, with most peaks occurring in promoters, introns, and distal intergenic regions. **C**, Principal component analysis of DARs from non-transformed BEAS-2B cells (NT, green); non-transformed cells that were transfected with an empty vector (EV, red); non-transformed cells expressing *SATB2* (SATB2, green); and iAs-transformed BEAS-2B cells (rapid transformation model; iAsT, purple). **D**, Overlapping transcription factor motifs between BEAS-2B cells expressing *SATB2* (SATB2) and iAs-transformed BEAS-2B cells (rapid transformation model). **E**, Top enriched *de novo* transcription factor motifs and best matching transcription factors in opened and closed DARs from BEAS-2B cells expressing *SATB2*. **F**, Congruence between DARs and proximal gene expression in BEAS-2B cells expressing *SATB2*. RNA UP = upregulated transcripts; RNA DN = downregulated transcripts. Counts are the number of individual genes above the minimum fold-change threshold for mRNA expression (> 1 Log_2_(FC)) or open chromatin (> 1 ShrunkenLog_2_(FC)). **G**, Top enriched C6 oncogenic signatures in non-transformed BEAS-2B cells that express *SATB2*. **H**, GSEA plot shows that genes upregulated in the KRAS pathway in iAsT cells were also enriched in cells expressing *SATB2*, while downregulated genes were similarly downregulated. **I**, In sh*SATB2*-KD cells, the enrichment in panel **I** is reversed; previously upregulated genes in the KRAS pathway are downregulated, and previously downregulated genes are upregulated

SATB2 organizes chromatin by binding to AT-rich cis-regulatory elements and associating with other SATB2 molecules and the inner nuclear matrix, thus facilitating long-range DNA interactions [32–34, 42]. To test for SATB2-associated chromatin changes, we performed ATAC-seq with the same lentiviral constructs and transformed BEAS-2B cells from above. We found that *SATB2* expression was associated with increased accessibility at 19,383 genomic regions and decreased accessibility at 14,014 others (Fig. 7A, Table S10), with clear distinctions within the top 50 differentially accessible peaks (Fig. 7B). Principal component analysis of DARs in non-treated, iAs-transformed, lentivirus-transformed, and SATB2-expressing BEAS-2B cells (Fig. 7C) suggest that a significant fraction of the DARs in iAs-transformed cells could be attributed to SATB2. Like the differentially accessible regions in iAs-transformed cells (Fig. 2 and S3), most of the SATB2*-* -associated DARs were found in distal enhancer-like regions, but there were no obvious differences in the genomic distribution of opened/closed DARs or ENCODE cCRE signatures (Fig S7A). Motif analysis of the SATB2-associated DARs identified 221 enriched transcription factor binding sequences (Benjamini *q*-value < 0.01), with 116 enriched in open chromatin and 105 in closed chromatin regions. There was a surprising number of transcription factor binding motifs that were shared between SATB2-expressing and iAs-transformed cells, including the NFE2L2 binding site (Fig. 7D). *De novo* motif discovery identified 12 binding sequences significantly enriched in open chromatin regions (*p* < 10^–11^), including binding sites for BATF, TEAD3, ERG, c-JUN, and AP2 (Fig. 7E), all transcription factors with established roles in oncogenesis [104–108]. Conversely, regions with reduced accessibility were significantly enriched for 13 *de novo* binding sequences, including the NFY/CCAAT promoter, SP2, AP-1 ELF1, and YY2 (Fig. 7E; Table S11), a pattern also observed in closed chromatin from iAs-transformed cells (Fig. 2E). Thus, SATB2 expression is associated with changes in accessibility at regions enriched for the CCAAT/enhancer binding protein (C/EBP) family of bZIP transcription factors that govern cell proliferation, apoptosis, and stress response [109–111].

We next asked if SATB2-dependent changes in chromatin accessibility are associated with changes in gene expression. We again annotated DARs to their nearest genes and compared proximal gene expression to (open vs. closed) chromatin state. Of the 2643 opened DARs, 660 proximal genes (25%) were upregulated; of the 986 closed DARs, 342 proximal genes (35%) were downregulated (Fig. 7F). The seemingly low correlation between DAR proximity and gene expression may be due to gene regulation by distal enhancer elements that are spatiotemporally arranged across chromosomes [112]. This hypothesis is supported by altered accessibility at ENCODE enhancer signatures and CCAAT enhancer binding sites in SATB2-expressing cells and iAs-transformed cells (e.g., Figs. 2E and 7E). It is also possible that nearby genes are regulated by repressors or silencers, DNA methylation status, or transcription factor access and timing [113, 114].

Next, we performed GSEA on the SATB2-dependent DARs and DEGs. Among 79 enriched oncogenic signatures in DEGs (Table S12), eight upregulated signatures occurred within newly opened chromatin, including KRAS signatures (Fig. 7G, Fig S7B, C) originally identified in iAs-transformed cells (Fig. 1D). We also saw increased pMEK levels in iAs-transformed and SATB2-expressing cells (see Fig. 6H), indicating an activated MAPK/ERK pathway (a downstream effector of KRAS signaling) that is associated with cell proliferation and oncogenic transformation. Notably, pERK was only upregulated in iAs-transformed cells and NF-κB expression was reduced in iAs-transformed cells and SATB2-expression conditions (Fig. 6H), suggesting that iAs-induced KRAS signaling proceeds through the MAPK pathway. Gene set enrichment analysis (GSEA) of the *SATB2-*expressing cells also showed enrichment for the NFE2L2-like oncogenic signature (Fig S7D), and other pathways (Fig S7E) that were first observed in iAs-transformed cells. When we performed GSEA on the SATB2 ranked gene set vs. the iAs-transformed ranked gene set, *SATB2* expression in non-treated cells showed positive enrichment for upregulated and downregulated genes in iAs-transformed cells (Fig. 7H). Further, knocking down *SATB2* (via shRNA) resulted in negative enrichment of those genes that were upregulated (Fig. 7I, top panel) and downregulated (Fig. 7I, bottom panel) gene sets in the iAs-transformed cells. These data show that SATB2 regulates these pathways in iAs-transformed cells, and that knocking down SATB2 reverses the gene expression profiles. Thus, SATB2-dependent changes in chromatin accessibility are responsible for a significant portion of the KRAS-like signatures found in iAs-transformed cells.

### *SATB2* and *circ3915* knockdown in transformed cells reverse oncogenic gene expression programs

**Fig. 8:**
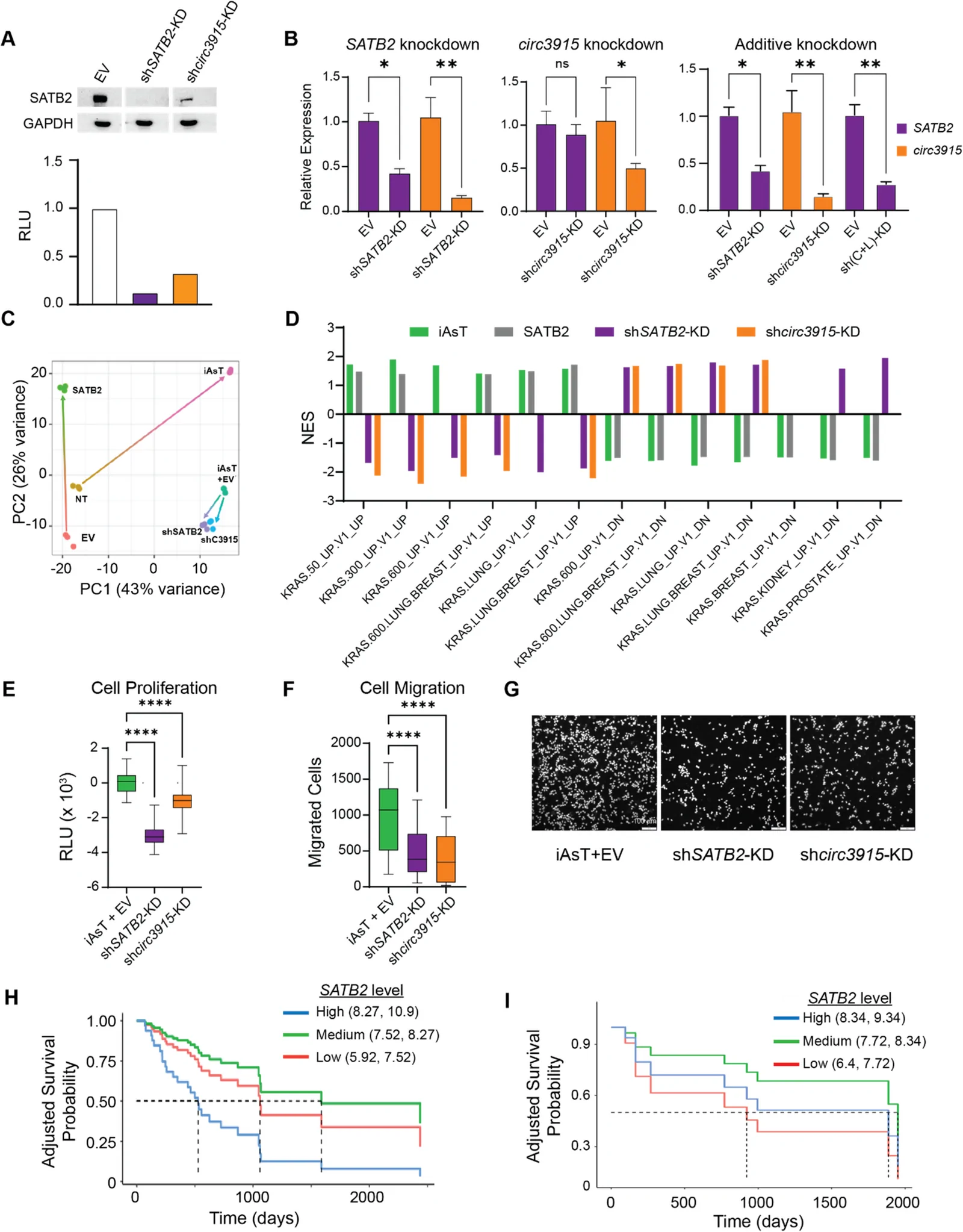
Knocking down *circ3915* or *SATB2* in iAs-transformed BEAS-2B cells reverses the *KRAS*-like gene signature. **A**, Western blot and GAPDH-normalized SATB2 expression in iAs-transformed BEAS-2B cells with stable *circ3915* or *SATB2* knockdown. EV = empty vector. **B**, Relative expression levels following shRNA-mediated knockdown of *SATB2*, *circ3915*, or combined knockdown using a probe (see Fig S1B) that targets both transcripts (C + L) compared with empty vector (EV) controls. Data are the mean ± s.d. from *n* = 3 replicates, as measured by RT-qPCR. EV = empty vector; ns = not statistically significant; * *p* < 0.05; ** *p* < 0.01. **C**, Principal component analysis (left) of RNA-seq expression profiles from non-transformed BEAS-2B cells (NT; brown); non-transformed cells containing an empty lentiviral expression vector (EV; red); non-transformed cells expressing *SATB2* (SATB2, green); iAs-transformed cells (iAsT; pink); iAs-transformed cells containing an empty lentiviral vector (iAsT + EV; turquoise); iAsT cells with stable *SATB2* knockdown (shSATB2; purple); and iAsT cells with stable *circ3915* knockdown (shC3915; light blue). iAs-transformed cells were generated with the rapid (12 week) transformation model. Arrows indicate how RNA expression profiles shifted from control samples to the matching experimental group. **D**, Bar plot of normalized enrichment scores for significantly enriched (*p*_*adj*_ <0.05) MSigDB oncogenic signatures in iAs-transformed BEAS-2B cells (iAsT, rapid transformation model; green), non-transformed cells expressing SATB2 (SATB2; grey), BEAS-2B cells with stable *SATB2* knockdown (sh*SATB2*-KD; orange), and BEAS-2B cells with stable *circ3915* knockdown (sh*circ3915*-KD; purple). **E**, iAs-transformed BEAS-2B cell proliferation after shRNA knockdown, as measured with a CellTiter-Glo ATP assay 72 h after seeding. iAsT + EV = iAs-transformed cells containing an empty lentivirus vector. RLU = relative light units. Luminescence was normalized to background, and values calculated relative to the control cells. N = three independent experiments with six technical replicates each; **** *p* < 0.0001. **F**, iAs-transformed BEAS-2B cell migration after shRNA knockdown, as measured by a transwell migration assay 24 h after seeding. Cells were fixed and stained with DAPI, and cell counts obtained with an Olympus cellSens counting module. Cell counts were normalized to the control cells. N = three independent experiments with three technical replicates each **** *p* < 0.0001. **G**, Representative images from the transwell migration assay of panel G. Scale bars = 100 μm. **H**,** I**. Kaplan-Meier plots of overall survival relative to *SATB2* expression in patients that either did not have a *KRAS* mutation (**H**, *n* = 342) or harbored a *KRAS* mutation (**I**, *n* = 150). Patient data were controlled for age at diagnosis, sex, and pack years

Having demonstrated that *SATB2* (and *circ3915*) expression is sufficient to activate KRAS-like gene expression patterns in non-transformed BEAS-2B cells, we asked if knocking down *SATB2* and/or *circ3915* in iAs-transformed cells could reverse these signatures and gene expression patterns. We transfected iAs-transformed cells with lentiviral vectors containing *SATB2*- and *circ3915*-targeted shRNAs, and then performed RNA-seq. Successful knockdown was confirmed by western blots and qRT-PCR (Fig. 8A, B). Knocking down *SATB2* mRNA affected 4421 genes, and knocking down *circ3915* affected 4085 genes (*p*_*adj*_. ≤ 0.05; Fig S8A).

Principal component analysis (Fig. 8C) showed that *SATB2-* and *circ3915*-knockdown cells clustered together, indicating that the two transcripts regulate similar genes (as expected).

Knockdown also shifted gene expression patterns away from *SATB2*-expressing cells (PC1) and iAs-transformed cells (PC2), suggesting a subset of gene expression changes depend on (but are not directly driven by) *SATB2* expression. GSEA confirmed that simultaneous *SATB2* and *circ3915* knockdown silenced the oncogenic signatures and pathways identified in iAs-transformed and *SATB2*-expressing cells, including the KRAS and NFE2L2 signatures (Fig. 8D, Fig S8B-D). Knocking down either transcript also decreased the proliferative and migratory capacity of iAs-transformed cells (Fig. 8E-G).

Because *SATB2* upregulation is associated with lung cancers (Fig. 3G) in the TCGA database [115], we next asked if *SATB2* expression is associated with survival. Reasoning that *SATB2* expression might be driving a *KRAS*-like phenotype, we separated tumor samples (from the TCGA database) into those with (*n* = 150) or without (*n* = 342) a *KRAS* mutation. We then used differential expression analysis (|Log_2_(FC)| > 1, *p* < 0.01) and Cox proportional hazard methods to control for *SATB2* expression, age at diagnosis, gender, race (filtered for White and Black/African American individuals due to limited data availability for other groups), and smoking status (calculated in pack years). We then censored for long-term survival by filtering individuals who survived at least two years and tracking them until the last follow-up date. When controlled for these factors, *SATB2* expression was associated with poor long-term survival in patients who did not harbor a *KRAS* mutation (HR = 1.85, *p* < 0.005; Fig. 8H), but not for patients who did have a *KRAS* mutation (HR = 0.979, *p* = 0.968; Fig. 8I). These data support the hypothesis that *SATB2* over-expression mimics a *KRAS*-like signature, and that *SATB2* expression is not necessary if a *KRAS* mutation is present.

## Discussion

### SATB2 produces a novel *circ3915* RNA that may participate in post-transcriptional regulation of SATB2 expression

In this study, we identify a previously uncharacterized *SATB2*-derived circular RNA (*circ3915*) that is co-expressed with SATB2 during iAs exposure and is detectable in human lung tumors. Messenger RNA and western blot data show that *circ3915* helps maintain the linear *SATB2* transcript, and anti-sense oligonucleotide data suggest that *circ3915* interacts with candidate SATB2-targeted miRNAs that are implicated in lung cancer cell proliferation, migration, and invasion [49]. Future studies will determine which of these candidate, full-length miRNAs are sequestered by *circ3915*. Nevertheless, these data are consistent with prior studies showing that *circSATB2* (distinct from *circ3915*) modulates lung cancer phenotypes by interacting with SATB2-targeted miRNAs [48–50, 116], and suggest that *circ3915*-mediated regulation of SATB2 likely involves post-transcriptional mechanisms.

We also provide multiple lines of evidence that *circ3915* can be translated by lung epithelial cells. Using tagged constructs, polysome association, puromycin sensitivity, and imaging approaches, we show that *circ3915* can give rise to a peptide (circ3915p) that localizes around the nucleus and appears to co-localize with SATB2. Proteomic analyses indicate that circ3915p can associate with SATB2-containing protein complexes and nuclear factors, including chromatin remodelers and transcription-associated proteins. While these findings suggest that circ3915p may have a functional role during iAs-induced cell transformation, detecting an endogenous peptide is technically challenging. Thus, further studies will be required to establish the presence and functional role of the endogenous circ3915 peptide.

### SATB2 expression is associated with altered chromatin accessibility adjacent to known oncogenes and *KRAS*-related transcription factors

SATB2 is a chromatin organizer, and in the BEAS-2B system, *SATB2* expression is associated with widespread changes in chromatin accessibility. iAs exposure induced substantial changes in chromatin accessibility at promoters, regulatory elements, and transcription factor binding sites, and genes proximal to these differentially accessible regions (DARs) were enriched for pathways related to epithelial-to-mesenchymal transitions, KRAS/EGFR signaling, and oxidative stress responses (NFE2L2).

Motif analysis revealed that regions with increased accessibility were enriched for transcription factor binding sites associated with KRAS-related signaling, whereas regions with decreased accessibility were enriched for motifs corresponding to CCAAT/enhancer binding protein (C/EBP) family members involved in proliferation and stress response [109–111]. Together, these findings indicate that SATB2 expression is associated with large-scale chromatin remodeling and transcriptional reprogramming linked to oncogenic pathways. Because these analyses are based on chromatin accessibility, however, they do not directly demonstrate SATB2 occupancy at specific cis-regulatory elements. Thus, the observed changes are best interpreted as SATB2-associated chromatin remodeling rather than direct targeting of specific loci. Future studies using ChIP-seq or related approaches will be required to determine if SATB2 directly binds to regulatory elements involved in these transcriptional programs.

### The SATB2/circ3915 axis regulates two oncogenic gene expression programs

*KRAS* mutations are present in approximately 30% of all lung cancers [117, 118], and *SATB2* and *circ3915* expression is associated with gene expression changes that resemble KRAS- and NFE2L2-like transcriptional programs. The NFE2L2 result is notable given that NFE2L2 is a master regulator of oxidative stress responses [119] and contribute to cancer cell survival [36, 120]. These patterns were observed in iAs-exposed cells and cells ectopically expressing *SATB2* and are attenuated when *SATB2* or *circ3915* are knocked down. Knockdown of *SATB2* or *circ3915* reduced proliferation and migration, indicating that both factors are required to sustain these cellular phenotypes. Interestingly, *circ3915* over-expression alone was sufficient to increase proliferation and migration in non-treated cells lacking detectable *SATB2* expression, indicating that *circ3915* may also function through additional regulatory mechanisms (e.g., RNA-binding proteins, candidate miRNAs, or translation into a peptide). Because *SATB2* over-expression generates *circ3915*, however, the gain-of-function phenotypes reflect the combined activity of SATB2 and *circ3915*, and do not fully distinguish SATB2-specific effects from *circ3915*-dependent contributions. Nevertheless, our findings indicate that a SATB2/*circ3915* regulatory axis contributes to iAs-induced, oncogenic transcriptional reprogramming.

### A proposed model, and potential implications

Chronic iAs exposure causes cancer [121–127], as does *SATB2* over-expression [45, 48–51] and the production of *SATB2* circular RNAs [48, 116, 128]. SATB2 and *SATB2*-derived circular RNAs are also implicated in lung cancer biology, although their roles appear to be context-dependent. For example, some studies show that reduced *SATB2* expression promotes EMT and invasiveness in NSCLC [129, 130], while others show that increased *SATB2* expression increases anchorage-independent growth and cell migration in human bronchial and renal cells [36, 41, 47, 131]. Thus, SATB2 function and its role in tumorigenesis may vary across environmental and cellular contexts.

The system described here provides a useful model for understanding how environmental exposures and chromatin organization regulate gene expression, independent of DNA mutations.

Our findings support a model in which iAs exposure induces *SATB2* and *circ3915* expression in lung epithelial cells, which then alters chromatin accessibility and reprograms oncogenic transcriptional networks. *circ3915* contributes to SATB2 mRNA stability and may participate in peptide-mediated interactions with chromatin-associated complexes. Patient datasets indicate that *SATB2* expression is associated with poor survival when tumors lack *KRAS* mutations, which is consistent with a potential link between *SATB2* expression and activated KRAS-like transcriptional programs. Future studies will be required to determine if SATB2 binds at or near oncogenic loci, and if an endogenous circ3915 peptide has a tumorigenic role. And while it is still unclear if SATB2 or *circ3915* are useful therapeutic targets, our findings suggest that this regulatory axis may be relevant in those cancers with transcriptional reprogramming but no obvious (or canonical) driver mutations. Targeting downstream pathways linked to these gene expression signatures might therefore provide therapeutic benefit in selected contexts.

## Supplementary Information

Supplementary Material 1: Table S1. siRNA sequences for knocking down SATB2 and circ3915 RNAs. Table S2. shRNA sequences for stable SATB2 RNA knockdown. Table S3. RT-qPCR primers for SATB2 and circ3915. Table S4. NanoString codesets for verifying differentially expressed circular and linear transcripts. Table S5. Excel file of differentially expressed genes in iAs-transformed BEAS-2B cells. Table S6. Excel file of differentially accessible regions in iAs-transformed BEAS-2B cells. Table S7. Excel file(s) of transcription factor binding sites/motifs in iAs-transformed BEAS-2B cells. Table S8. Protein-protein interactions in BEAS-2B cells expressing SATB2 or cric3915p. Table S9. Excel file of dysregulated genes in non-transformed BEAS-2B cells that express SATB2. Table S10. Excel file of differentially accessible regions in non-transformed BEAS-2B cells that express SATB2. Table S11. Excel file(s) of transcription factor binding sites/motifs in non-transformed BEAS-2B cells that express SATB2. Table S12. Enriched gene sets in SATB2-dependent DARs and DEGs.

Supplementary Material 2: Fig S1. General experimental methods. A, General location of ON-TARGETplus small interfering (si) RNAs for transiently knocking down SATB2 mRNA or circ3915. Numbers correspond to SATB2 exons. C+L = probe for circular and linear RNA. B, General location of Millipore Sigma short hairpin (sh) RNAs for stably knocking down SATB2 mRNA or circ3915. C, Cloning strategy for expressing SATB2 mRNA (top) or circ3915 (bottom) from the pcDNA3.1(+) vector in non-transformed BEAS-2B cells. Once transcribed, the inverted Alu repeats in the pre-mRNA form a loop that promotes back-splicing. D, Position of the tag (HiBiT or 3×FLAG) in the lentiviral circ3915 expression vectors. The tag is placed upstream of the putative start codon to ensure that any peptide is derived from the back-spliced transcript. E, RT-qPCR primer design strategy for specifically detecting linear SATB2 mRNA, circ3915, or both transcripts. F, NanoString probe design for detecting linear (top, red fluor) and circular (bottom, green fluor) RNAs. RNA isoforms are counted with complementary capture and reporter probes designed to hybridize to the linear splice and circular back-splice junctions. RNA isoforms were counted via a unique barcode assigned to each reporter probe. Images created with BioRender. Fig S2. Inorganic arsenic (iAs) exposure promotes transformation by driving oncogenic gene expression programs. Data were generated from the rapid (12-week) exposure model. A, Principal component analysis of RNA-seq expression profiles from non-transformed (NT, pink) and iAs-transformed BEAS-2B cells (iAsT, blue). B, The top 30 differentially expressed genes (DEGs) in iAs-transformed (iAsT) cells relative to non-transformed (NT) controls. C, Correlation between RNA-seq and NanoString gene expression levels for 191 DEGs. Dashed lines = 95% confidence interval. D, The top enriched biological processes reflected in the iAsT DEGs. E, The top enriched KEGG pathways in the iAsT DEGs. F, The top enriched Reactome pathways in the iAsT DEGs. G, The top enriched MSigDB hallmarks in the iAsT DEGs. Fig S3. Inorganic arsenic induces global changes in chromatin accessibility. iAs-transformed cells were generated with the rapid (12-week) exposure model. A, DARs between non-transformed (NT) and iAs-transformed (iAsT) cells. B, Principal component analysis of differentially accessible regions in non-transformed (NT, black) and iAs-transformed (brown, iAsT) BEAS-2B cells. C, Genomic distance between ATAC-seq peaks and promoters (left) or gene bodies (right) in iAsT DARs. D, Congruence between genes associated with open chromatin and closed chromatin in iAsT cells. E, The top enriched biological processes reflected in the iAsT DARs. F, The top enriched KEGG pathways in the iAsT DARs. G, The top enriched Reactome pathways in the iAsT DARs. H, The top enriched MSigDB hallmarks in the iAsT DARs. I, the top enriched C6 oncogenic signatures in the iAsT DARs. Fig S4. circRNA detection in iAs-transformed cells. A, Microarray-based detection of differential circRNA expression in pre-transformed (0.5 µM iAs for 17 weeks) and fully transformed (+ 2.0 µM iAs for another 10 weeks) BEAS-2B cells relative to non-treated cells. Upregulated circRNAs were defined by p < 0.05 and fold change (FC) >1.2. Downregulated circRNAs were defined by p <0.05 and FC < -1.2. B, Overlapping circRNA expression in pre-transformed and fully transformed BEAS-2B cells, as measured by Arraystar microarray. C, SATB2 mRNA and circ3915 expression in 16HBE cells after 48 hr exposure to increasing concentrations of iAs. D, SATB2 mRNA and circ3915 expression in 16HBE cells in the two-hit exposure model. * p < 0.05, *** p < 0.001, **** p < 0.00001. E, Validation of circ3915 using convergent and divergent primers. Primers were designed to amplify the backsplice junction (targeting exons 3-6; 211 bp) and a linear region common to SATB2 and circ3915 (Exon 3, 130 bp). Convergent primers amplify cDNA and gDNA, while divergent primers only amplify cDNA. The bands with diverging primers on cDNA were cut, purified, and sequenced to confirm the presence of the backsplice junction (chromatograms are shown above the agarose gel images). Fig S5. Predicted interactions between SATB2 and circ3915p. A, 3×FLAG (blue) tagged circ3915 expression vector showing the backsplice junction (yellow), and start codon (green). The predicted circ3915p amino acid sequence is shown in Fig 3B; note the numerous lysines (K) present in the backsplice junction. Peptides detected by LC-MS/MS are shown beneath the construct, with a representative mass spectrum shown in B. C, Puromycin treatment shifts endogenous circ3915 translation towards light and free polysomes. CHX = cycloheximide. D, In contrast, a known but untranslated endogenous circRNA (hsa_circ_0008928) does not associate with polysomes and does not show a shift in polysome association when treated with puromycin. E, Alphafold3 predicted structure and space-filling model for the SATB2-circ3915p complex (SATB2 UniProt accession Q9UPW6-1). F, Same as panel E, except color coding indicates the predicted local Distance Difference Test (plDDT), or the quality of the predicted interactions. Structured regions are reflected in blue, and disordered regions are reflected in orange. G, Same as panels E and F, except colors now reflect the SATB2 domains. The red square box is enlarged in panel H, and suggests that SATB2 and circ3915p interact through their respective ubiquitin-like domains. I, J. Western blots for SATB2-IP (I) and circ3915p (FLAG-IP; J), validating IP-MS data from Table S4. Primary antibodies are found in the Key Resources table. Fig S6. Differentially expressed genes (DEGs) in non-transformed BEAS-2B cells that express SATB2. A, Differentially expressed genes (DEGs) in non-transformed BEAS-2B cells that express SATB2 (SATB2). In total, 5178 genes were upregulated, and 5492 were downregulated at padj < 0.05. B, Top 30 differentially expressed genes from panel A. Control BEAS-2B cells contained an empty expression vector (EV). C, Correlation between RNA-seq and NanoString gene expression levels for 191 DEGs in non-transformed BEAS-2B cells that express SATB2. Dashed lines = 95% confidence interval; FC = fold-change D, Overlapping up- and downregulated genes in iAs-transformed (iAsT) BEAS-2B cells and non-transformed BEAS-2B cells expressing SATB2. iAsT cells were generated with the rapid (12-week) transformation model. Fig S7. Differentially accessible regions (DARs) in non-transformed BEAS-2B cells that express SATB2. A, Genomic distribution of DARs (top) and ENCODE candidate cis-regulatory elements (cCREs; bottom) in non-transformed BEAS-2B cells expressing SATB2. DARs were defined by FDR <0.01 and Shrunken Log2(FC) >1 for opened DARs, and Shrunken Log2(FC) <-1 for closed DARs. PLS = promoter-like signatures; pELS = proximal enhancer-like signatures; dELS = distal enhancer-like sequences. In total, there were 6963 opened chromatin regions, and 2089 closed regions. B, MSigDB oncogenic signatures in non-transformed BEAS-2B cells expressing SATB2 that were commonly enriched in DARs and DEGs. ATAC UP = opened chromatin; ATAC DN = closed chromatin; RNA UP = upregulated transcripts; RNA DN = downregulated transcripts. C, SATB2 opens chromatin at KRAS signature genes, increasing their gene expression. Shown is an integrative genomics viewer (IGV) snapshot of normalized average peak distributions for KRAS_UP_UP signature genes. NT = non-transformed BEAS-2B cells. iAsT = iAs-transformed BEAS-2B cells generated with the rapid (12-week) transformation model; EV = non-transformed BEAS-2B cells containing an empty expression vector; SATB2 = non-transformed BEAS-2B cells expressing SATB2. Encode cCREs are shown in yellow (dELSs), orange (pRLSs), and red (PLS). D, RNA expression of 161 genes from the MSigDB oncogenic NFE2L2 signature. E, Top enriched MSigDB Hallmark pathways in non-transformed BEAS-2B cells that express SATB2. Fig S8. Knocking down circ3915 or SATB2 in iAs-transformed BEAS-2B cells reverses oncogenic KRAS-like gene expression programs. A, Differentially expressed genes in iAs-transformed cells with stable SATB2 (shSATB2-KD) or circ3915 (shcirc3915-KD) knockdown. Upregulated RNAs were defined by padj < 0.05 and fold change (FC) >1.2, and downregulated RNAs were defined by padj < 0.05 and FC <-1.2. Blue dots represent RNAs with no significant difference in RNA expression. Knocking down SATB2 affected 4421 genes (2698 upregulated and 1723 downregulated), and knocking down circ3915 affected 4085 genes (1898 upregulated and 2187 downregulated). B, SATB2 (shSATB2) and circ3915 (shcirc3915) knockdown reverses MSigDB KRAS-like oncogenic programs/signatures seen in iAs-transformed (iAsT) BEAS-2B cells (rapid transformation model) and non-transformed BEAS-2B cells that express SATB2 (SATB2). Each row represents a KRAS signature gene from the indicated MSigDB oncogenic signature. C and D, Dot plots of GSEA-enriched MsigDB oncogenic signature gene sets in differentially expressed genes (padj < 0.05) from stable SATB2 (C) or circ3915 (D) knockdown in iAs-transformed cells show that KRAS-like and NFE2L2 MSigDB oncogenic gene expression signatures are reversed (suppressed).

Supplementary Material 3.

## Data Availability

Raw and analyzed data reported in this paper are available at the Gene Expression Omnibus as GSE278219 and are publicly available as of the date of publication. All data will be shared by the lead contact upon request. This paper does not report original code. Any additional information required to reanalyze the data reported in this paper is available from the lead contact upon request.
