# Supplementary material for: SATB2 dysregulation generates a novel circular RNA and drives KRAS-like transcriptional reprogramming and transformation-associated phenotypes
Source: Cell Commun Signal. 2026 Jun 24;24:476. doi: 10.1186/s12964-026-03021-9 (PMC13551775; doi:10.1186/s12964-026-03021-9)
Supplement: Supplementary file 1 [file 12964_2026_3021_MOESM1_ESM.docx]

**KEY RESOURCES TABLE**

| REAGENT or RESOURCE | SOURCE | IDENTIFIER |
| --- | --- | --- |
| Antibodies | | |
| SATB2, 0.1 mg/mL | Invitrogen | PA5-83092 |
| GAPDH, 0.94 mg/mL | Abcam | ab9485 |
| Anti-GFP 1:2000 | Abcam | Ab290 |
| Anti-mouse IgG, 0.2 mg/mL | Proteintech | SA00001-1 |
| Anti-rabbit IgG, 0.2 mg/mL | Proteintech | SA00001-2 |
| Anti-mouse Alexa Fluor 594, 2 mg/mL | Invitrogen | A-11005 |
| Epithelial-Mesenchymal Transition (EMT) Antibody Sampler Kit | Cell Signaling Technology | 9782 |
| p44/42 MAPK (Erk1/2) Antibody | Cell Signaling Technology | 9102 (Rb) |
| Phospho-p44/42 MAPK (Erk1/2) (Thr202/Tyr204) (D13.14.4E) XP® Rabbit mAb | Cell Signaling Technology | 4370 |
| NF-κB p65 XP mAb | Cell Signaling Technology | D14E12 |
| ERK1/2 Polyclonal antibody | Proteintech | 11257-1-AP |
| Phospho-ERK1/2 (Thr202/Tyr204) Recombinant antibody | Proteintech | 80031-1-RR |
| DDX17 | Proteintech | 19910-1-AP |
| Lamin | Invitrogen | MA5-35284 |
| SUPT16H | Invitrogen | MA5-27214 |
| H2A.Z | Invitrogen | 711485 |
| H2A.X (1H65L16) | Invitrogen | 701953 |
| H2A | Cell Signaling Technology | 12349 |
| H2B | Invitrogen | 710912 |
| FLAG M2 | Millipore Sigma | F1804/F3165 |
| Biological samples |  |  |
| Matched normal and tumor samples from lung adenocarcinoma and sarcoma patients | Van Andel Institute Pathology and Biorepository Core | RRID:SCR_022912 |
| Chemicals, peptides, and recombinant proteins | | |
| Dulbecco's Modified Eagle's Medium | Sigma-Aldrich | D7777 |
| Gibco Advanced Modified Eagle’s Medium | Thermo Fisher Scientific | 12492013 |
| 10% fetal bovine serum | Sigma-Aldrich | F0926 |
| Penicillin-streptomycin, 100 U/mL | Sigma-Aldrich | P4333 |
| GlutaMAX | Thermo Fisher Scientific | 35050079 |
| NaAsO_2_ | Fisher | S225I-100 |
| Lipofectamine 3000 reagent | Life Technologies | L3000015 |
| Opti-MEM™ I Reduced Serum Medium | Life Technologies | 31985070 |
| Polyethylenimine, 2500 mw | Polysciences, Inc. | 24313 |
| Trypsin | Thermo Fisher Scientific | 25200072 |
| Trypan blue | Thermo Fisher Scientific | 15250061 |
| Phosphate buffered saline (PBS) | Thermo Fisher Scientific | 10010023 |
| Nano-Glo® Luciferase Assay Reagent | Promega | N3030 |
| Bovine serum albumin (BSA) | Thermo Fisher Scientific | B14 |
| Collagen | Advanced BioMatrix | 5005-100ml |
| Epidermal growth factor (EGF) | Thermo Fisher Scientific | PHG0314 |
| Triton X-100 | Millipore-Sigma | X100 |
| DAPI | Sigma-Aldrich | D9542 |
| Puromycin | Sigma-Aldrich | P7225 |
| Cyclohexamide | Thermo Fisher Scientific | J66901.03 |
| HALT Protease Inhibitor Cocktail (100x) | Thermo Fisher Scientific | 78440 |
| RNAseOut | Invitrogen | 10777019 |
| 4x Laemmli Sample Buffer | Bio-Rad | 1610747 |
| β-mercaptoethanol | Bio-Rad | 1610710 |
| Sodium deoxycholate | Millipore Sigma | D1501 |
| TRIzol reagent | Invitrogen | 15596026 |
| DNAse I | Zymo Research | E1010 |
| Dulbecco’s Phosphate Buffered Saline (DPBS) | Sigma-Aldrich | D8537 |
| Radioimmunoprecipitation assay (RIPA) lysis and extraction buffer | Thermo Fisher Scientific | J62885.AE |
| Phenylmethylsulfonyl fluoride (PMSF) | Sigma-Aldrich | 10837091001 |
| 1X protease inhibitor cocktail | EpiGentek | R-1101 |
| 3-methoxybenzylamine (3-MBZ) | Sigma-Aldrich | 159891 |
| Benzylamine | Sigma-Aldrich | 185701 |
| ProLong Diamond Antifade Mountant | Invitrogen | P36965 |
| Actinomycin D | Sigma-Aldrich | A9415 |
| Critical commercial assays | | |
| Monarch PCR and DNA Purification Kit | New England Biolabs | T1030S |
| NEBuilder® HiFi DNA Assembly Cloning Kit | New England Biolabs | E5520S |
| CellTiter-Glo ATP Assay | Promega | G9241 |
| Quick-RNA Miniprep Kit | Zymo Research | R1054 |
| Arraystar Human Circular RNA Array | Arraystar | AS-S-CR-H-V2.0 |
| QIAseq FastSelect –rRNA HMR Kit | Qiagen | 334376 |
| KAPA RNA HyperPrep Kit | Roche | KK8580 |
| TruSeq UD Indexes adapters | lllumina |  |
| QuantiFluor® dsDNA System | Promega | E2671 |
| Kapa Illumina Library Quantification qPCR assay | Roche | KK4844 |
| nCounter™ SPRINT cartridge | NanoString Technologies | NAA-CART-048 |
| Nano-Glo HiBiT Blotting system (with HiBiT and LgBiT antibodies and reagents) | Promega | N2410 |
| Pierce BCA Protein Assay Kit | Pierce | 23225 |
| TBST blocking buffer | Fisher BioReagents | BP337-500 |
| Dyna Beads Protein G | Invitrogen | 10004D |
| Pierce IP Lysis Buffer | Thermo Fisher Scientific | 87787 |
| FLAG Immunoprecipitation Kit | Millipore Sigma | FLAGIPT1 |
| Dynabeads™ MyOne™ Streptavidin C1 | Thermo Fisher Scientific | 65002 |
| Lentiviral production, transduction, and selection | U. Michigan Vector Core Laboratory, Ann Arbor, MI | |
| Arraystar circRNA service | Arraystar, Rockville, MD | |
| ATAC-seq service | ActiveMotif, Carlsbad, CA | |
| Mass spectrometry service | Michigan State University Integrated Mass Spectrometry Unit, East Lansing, MI | |
| Deposited data | | |
| Human genome reference build 38 (hg38) | NIH, NCBI; https://www.ncbi.nlm.nih.gov/datasets/genome/GCF_000001405.26/ | |
| Uniprot Homo sapiens (UP000005460) database | EMBL-EBI; https://www.uniprot.org/proteomes/UP000005640 | |
| The Cancer Genome Atlas database | https://portal.gdc.cancer.gov/ | |
| GEO with accession numbers | GSE278219 | |
| Experimental models: Cell lines | | |
| BEAS-2B cells | ATCC | CRL-3588 |
| 16HBE cells | Dr. Shaun McCullough, EPA | |
| Oligonucleotides | | |
| ON-TARGETplus SATB2 siRNAs | Dharmacon Reagents | LQ-023161-01 |
| ON-TARGETplus Non-targeting Control siRNAs | Dharmacon Reagents | D-001810-01 |
| [UAAAGGUUUGAUGAUUCCUGUdTdT](http://blast.ncbi.nlm.nih.gov/Blast.cgi?PROGRAM=blastn&PAGE_TYPE=BlastSearch&LINK_LOC=blasthome&QUERY=%3Ehsa_circ_0003915-siRNA1%0ATAAAGGTTTGATGATTCCTGT&DATABASE=nr&EQ_MENU=Homo%C2%A0sapiens%C2%A0(taxid:9606)) | This study | circ3915-1 siRNA |
| [GAUUAAAGGUUUGAUGAUUCC](http://blast.ncbi.nlm.nih.gov/Blast.cgi?PROGRAM=blastn&PAGE_TYPE=BlastSearch&LINK_LOC=blasthome&QUERY=%3Ehsa_circ_0003915-siRNA2%0AGATTAAAGGTTTGATGATTCC&DATABASE=nr&EQ_MENU=Homo%C2%A0sapiens%C2%A0(taxid:9606))dTdT | This study | circ3915-2 siRNA |
| [UUAAAGGUUUGAUGAUUCCUG](http://blast.ncbi.nlm.nih.gov/Blast.cgi?PROGRAM=blastn&PAGE_TYPE=BlastSearch&LINK_LOC=blasthome&QUERY=%3Ehsa_circ_0003915-siRNA3%0ATTAAAGGTTTGATGATTCCTG&DATABASE=nr&EQ_MENU=Homo%C2%A0sapiens%C2%A0(taxid:9606))dTdT | This study | circ3915-3 siRNA |
| SATB2 shRNAs | Millipore Sigma | TRCN0000020685, TRCN0000020687, and TRCN0000020688 |
| [UAAAGGUUUGAUGAUUCCUGU](http://blast.ncbi.nlm.nih.gov/Blast.cgi?PROGRAM=blastn&PAGE_TYPE=BlastSearch&LINK_LOC=blasthome&QUERY=%3Ehsa_circ_0003915-siRNA1%0ATAAAGGTTTGATGATTCCTGT&DATABASE=nr&EQ_MENU=Homo%C2%A0sapiens%C2%A0(taxid:9606)) | This study | shcirc3915-1 |
| [GAUUAAAGGUUUGAUGAUUCC](http://blast.ncbi.nlm.nih.gov/Blast.cgi?PROGRAM=blastn&PAGE_TYPE=BlastSearch&LINK_LOC=blasthome&QUERY=%3Ehsa_circ_0003915-siRNA2%0AGATTAAAGGTTTGATGATTCC&DATABASE=nr&EQ_MENU=Homo%C2%A0sapiens%C2%A0(taxid:9606)) | This study | shcirc3915-2 |
| [UUAAAGGUUUGAUGAUUCCUG](http://blast.ncbi.nlm.nih.gov/Blast.cgi?PROGRAM=blastn&PAGE_TYPE=BlastSearch&LINK_LOC=blasthome&QUERY=%3Ehsa_circ_0003915-siRNA3%0ATTAAAGGTTTGATGATTCCTG&DATABASE=nr&EQ_MENU=Homo%C2%A0sapiens%C2%A0(taxid:9606)) | This study | shcirc3915-3 |
| ttggacggctctcttgaatatg | This study | SATB2 circular + linear forward primer |
| ccgcagagctgtgagaatac | This study | SATB2 circular + linear reverse primer |
| tctccccaaacacaccatca | This study | SATB2 linear mRNA forward primer |
| gcagctcctcgtccttatattca | This study | SATB2 linear mRNA reverse primer |
| gaagattaaaggtttgatgattcctgtc | This study | circ3915 forward primer |
| ccaccttcccagcttgattatt | This study | circ3915 reverse primer |
| gtcagccgcatcttcttttg | This study | GAPDH forward primer |
| gcgcccaatacgaccaaatc | This study | GAPDH reverse primer |
| gcaccacgtccaatgacat | This study | RPII forward primer |
| gtgcggctgcttccataa | This study | RPII reverse primer |
| ttcggagagttctgggattgta | This study | TBP forward primer |
| tggactgttcttcacttcttggc | This study | TBP reverse primer |
| GACAGGAATCATCAAACCTTTAATCTTC-Biotin-TEG | This study | circ3915 antisense oligonucleotide (ASO) |
| TTAGTCGACATGTAAACCA-Biotin-TEG | This study | Nonsense ASO oligonucleotide (negative control) |
| Recombinant DNA | | |
| Lentivirus U5 promoter-shRNA; CMV-GFP vector | U. Michigan Vector Core Laboratory | |
| pcDNA3.1(+) CircRNA Mini Vector | Addgene | 60648 |
| SATB2-GFP circ3×FLAG-tagged construct | This study | |
| SATB2-GFP construct | This study | |
| circ3915 3×FLAG-tagged construct | This study | |
| circ3915 HiBiT construct | This study | |
| psPAX2 | U. Michigan Vector Core Laboratory | |
| pC1-VSVG | U. Michigan Vector Core Laboratory | |
| Software and algorithms | | |
| Circular RNA interactome resource | NIH, NIA; https://circinteractome.nia.nih.gov/index.html | |
| multiMiR R package | Ru et al. 2014(83) | |
| RTA3 | Illumina | |
| Bcl2fastq v2.20.0 | Illumina | |
| TrimGalore v0.6.10 | doi.org/10.5281/zenodo.7598955; https://github.com/FelixKrueger/TrimGalore | |
| STAR v2.7.10a | Dobin et al. 2013(66) | |
| DESeq2 v1.38.3 | Love et al. 2014(67) | |
| clusterProfiler v4.6.2 | Wu et al. 2021(69) | |
| msigdbr v7.5.1 | https://software.broadinstitute.org/cancer/software/gsea/wiki/index.php/MSigDB_v7.5.1_Release_Notes | |
| enrichPlot v1.18.4 | DOI: 10.18129/B9.bioc.enrichplot | |
| ROSALIND® | https://rosalind.onramp.bio/ | |
| geNorm | Perkins et al. 2012(70) | |
| skewer v.0.2.2 | Jiang et al. 2014(71) | |
| Samtools v0.1.19 | Danecek et al. 2021(72) | |
| BWA algorithm | Li H. and Durbin R. 2010(73) | |
| deeptools v3.5.1 | Ramírez et al. 2016(74) | |
| MACS 2.1.0 | Zhang et al. 2008(75) | |
| ChIPseeker v1.34.1 | Yu et al. 2015(78) | |
| GenomicRanges v1.50.2 | Lawrence et al. 2013(79) | |
| Statistical software | GraphPad Prism version 10.0.0 for Windows | |
| BioRender | https://BioRender.com | |
| Other | | |
| 0.45 µm HV-Durapore Stericup | Millipore Sigma | S2HVU05RE |
| ThinCert trans-well inserts, 8 µm pore | Greiner Bio-One | 662638 |
| DNA High Sensitivity chips | Agilent | 5067-4626 |
| Cell culture flasks | Nest Scientific | 708001 |
| SuperSignal West Dura Extended Duration Substrate | Thermo Fisher Scientific | 34075 |
