# Supplementary material for: SATB2 dysregulation generates a novel circular RNA and drives KRAS-like transcriptional reprogramming and transformation-associated phenotypes
Source: Cell Commun Signal. 2026 Jun 24;24:476. doi: 10.1186/s12964-026-03021-9 (PMC13551775; doi:10.1186/s12964-026-03021-9)
Supplement: Supplementary file 2 [file 12964_2026_3021_MOESM2_ESM.docx]

**Fig S1**

**Fig S2**

.

**Fig S3**

**Fig S4**

**Fig S5**

**Fig S6**

**Fig S7**

**Fig S8**
